# The ARCH model: a neuroevolutionary framework for behavioral execution

**DOI:** 10.3389/fpsyt.2025.1669530

**Published:** 2025-10-13

**Authors:** Tahir Rahman, Charles F. Zorumski, J. Reid Meloy

**Affiliations:** ^1^ Department of Psychiatry, Washington University in St. Louis, St. Louis, MO, United States; ^2^ San Diego Psychoanalytic Center, San Diego, CA, United States

**Keywords:** constraint-convergent behavior, neuroethology, cultural cognition, behavioral systems neuroscience, motivational drives, archetypes, ARCH equation, computational psychiatry

## Abstract

Behavior arises from the convergence of multiple constraints rather than single causes. The ARCH × Φ model formalizes this process as a computational grammar of behavior, in which Archetype (A), Drive (D), and Culture (C) interact multiplicatively, and expression occurs only when a context-sensitive threshold (Φ) is crossed. This scalar–vector framework specifies behavior as probabilistic and testable, supporting hypotheses that can be evaluated across neurobiological, behavioral, and symbolic domains. We define a provisional taxonomy of ten archetypal systems (Systema Behavorum), such as Agonix (competition), Theromata (caregiving), and Sacrifex (self-sacrifice), which serve as structured inputs to the grammar. ARCH × Φ integrates ethology, affective neuroscience, psychiatry, and cultural psychology, reframing archetypes not as metaphors but as conserved neural scripts subject to scalar amplification and symbolic modulation. The framework supports falsifiable predictions, operational definitions, and clinical applications in decoding motivation, threshold dysregulation, and symbolic distortion. ARCH × Φ thus reframes behavior as an emergent property of convergent constraints across biology, affect, culture, and context.

"What begins as a conserved neural script becomes behavior when Drive energizes it, Culture assigns salience, and the threshold (Φ) is crossed."

## 1 Introduction

The convergence of phylogenetics, evolutionary theory, and neuroscience offers a robust framework for understanding the origins of behavior. Across species, recurrent behavioral patterns—ranging from caregiving to territorial aggression—reflect deeply conserved architectures sculpted by natural selection. The quest to identify lawful principles that govern such patterns has long engaged disciplines as diverse as ethology, affective neuroscience, psychology, psychiatry, and anthropology (1–3). Lewin's field theory (1936) was an early attempt to formalize behavior as the product of interacting forces within a dynamic psychological space, expressed as B = f(P, E), where B is behavior, P the person, and E the environment (4). Psychological forces were treated as vectors with direction and magnitude, extending Gestalt psychology's search for lawful principles of organization. Lewin sought to model how internal and external forces combine into coherent behavioral wholes, initiating the quantitative study of behavior as the dynamic integration of parts into structured systems.

Recent computational work confirms that Gestalt principles (such as the laws of similarity, prägnanz, proximity, and closure) can be formalized and quantified. Neural networks trained on natural images exhibit closure effects when tested with fragmentary stimuli, showing representational similarity between aligned fragments and complete figures, but not with disordered fragments (5). For example, when partial arcs are arranged to suggest a circle, both humans and trained networks "close the gap" and perceive a complete circular figure, whereas the same arcs rearranged randomly do not produce closure. These findings suggest that coherence emerges only when partial inputs converge under conjunctive, threshold-dependent rules, extending Gestalt insights into modern computational neuroscience.

Building on evolutionary logic, E.O. Wilson's sociobiological synthesis posited that inherited predispositions and modular subsystems organize species-typical behaviors through selective interactions with ecological niches (6). More recently, Friston's free-energy principle advances a formal, thermodynamic interpretation: biological systems act to minimize the divergence between internal generative models and external sensory input, thereby reducing entropy. Under this model, behavior is conditional, emerging only when multiple internal and environmental constraints converge to resolve uncertainty (7, 8). Taken together, these traditions illustrate a recurring challenge: while prior models have described behavior as the interplay of internal and external forces, none have provided a unified grammar capable of spanning evolutionary inheritance, motivational energy, and cultural meaning. Gestalt psychology clarified how partial inputs can cohere into structured wholes, while sociobiology emphasized inherited predispositions, and predictive coding formalized the minimization of uncertainty. However, each remains partial. What is needed is a framework that specifies not only the structural templates of behavior, but also the energetic forces that activate them and the symbolic contexts that shape their trajectory. The ARCH model was developed to meet this need by providing a unified grammar of behavior. In subsequent sections, we formalize this grammar into a computational equation, ARCH × Φ, that makes its predictions explicit and testable. This constraint-satisfaction framework underlies our ARCH model (9), in which behavior emerges not from singular causes, but from the confluence of three independently necessary factors: *Archetypes* (A), *Drives* (D), and *Culture* (C). These factors interact multiplicatively, such that the absence of any single component suppresses behavioral expression (1 × 1 × 0 = 0). In its initial forensic application, we formulated the ARCH model as:


Behavior=A×D×C

Here, archetypes were conceptualized as evolutionarily conserved neural scripts—modular systems of perception, motivation, and behavior instantiated in brain circuitry (9–12). These templates are latent, emotionally valenced, and often symbolically encoded; their activation depends on the convergence of internal motivational states and external cues. Jung referred to them as instinctual drives present in all living things (10). Like fixed action patterns in ethology—such as Tinbergen's classic finding that male stickleback fish reliably attacked crude models with red undersides—archetypes structure the form of behavior but remain dynamically modulated by context, learning, and symbolic framing. Established ethological research demonstrates how exaggerated, or *supernormal stimuli*—such as oversized artificial eggs that elicit stronger retrieval than natural ones^1^—can hyperactivate conserved behavioral programs (13). In humans, analogous cultural cues may similarly distort or amplify archetypal expression, producing what we have termed *Fixed Archetypal Action Patterns* (FAAPs) (9). These two concepts will be described in further detail in sections 8.0 and 9.0.

Complementing these are biological drives—such as hunger, fear, sexual arousal, and the pursuit of recognition—that supply the energetic momentum for action. These systems are deeply embedded in subcortical circuitry, shaped by neuromodulators such as dopamine, oxytocin, serotonin, and testosterone (14). In humans, these drives are rarely expressed in their raw form; instead, they are shaped, inhibited, or attenuated by cultural systems—the social, symbolic, and normative structures that endow behavior with meaning and direction. Archetypes may thus be instantiated neurobiologically (e.g., parental care via the medial preoptic area) (10, 12, 15), but they are often expressed culturally as monastic service, clinical caregiving, or ideological sacrifice. The competitive archetype may emerge as physical aggression in one context and academic perfectionism in another.

Culture is not an overlay, it is a co-constructive domain that sculpts symbolic expression and behavioral cues from conserved neural grammars (10, 16–19). For example, the same female rodent—depending on the cue—may display lordosis in response to a male conspecific or a startle/freeze response when confronted with a predator, such as a snake (20, 21). Taken together, the interaction of these domains yields a biologically grounded equation of behavior: conserved neural structure (A), energized by internal drives (D), and sculpted by cultural framing (C). This triadic system offers a middle path between strict biological determinism and social constructivism—what we refer to as *constraint within possibility*.

Yet this original equation is incomplete. First, it lacks a mechanism to model *activation thresholds*, that is, the minimum conditions under which latent neural scripts cross into overt behavior. Second, it fails to capture the *directionality* and *compositionality* of archetypes. Archetypes are not scalar values but structured vectors—multi-dimensional templates such as *Warrior*, *Martyr*, *Healer*, or *Avenger*—each specifying a unique behavioral trajectory. Accordingly, we extend the model into a higher-order formulation: the ARCH × Φ equation.

In this formulation:

- A (Archetype): a vector representing evolutionarily conserved neurocircuit scripts that structure perception, affect, and action.
- D (Drive): a scalar representing biologically instantiated motivational energy, amplified or attenuated by neuromodulators and endocrine states.
- C (Culture): a scalar representing symbolically coded cues, ranging from immediate triggers to collective meaning systems, that bias archetypal activation.
- Φ (Threshold): a context-sensitive gating field that regulates whether latent scripts cross into behavioral expression.

This scalar–vector logic allows us to model behavior as both structured and context-sensitive. It enables composite archetypal activations (e.g., *Hero + Martyr*), the modulation of intensity (via *D* and *C*), and threshold-dependent activation (*Φ*), consistent with modern neuroscience and systems biology. Moreover, it accommodates a wide range of behaviors—from reflexive action to symbolic self-sacrifice—within a unified explanatory grammar (22–35). This vector-based model builds on the foundational insights of Lewin, who first conceptualized behavior as the result of directional psychological forces within a structured field (4). However, Lewin's original formulation lacked a mechanism for activation thresholds or the compositional logic of archetypal systems. Contemporary models in neuroscience and behavioral science—including affective systems theory (Panksepp), predictive coding (Friston), and attractor dynamics in decision theory—extend Lewin's original vector logic by incorporating thresholds, feedback loops, and multidimensional constraint spaces (3, 7, 12).

The ARCH × Φ model builds upon and extends prior frameworks of biological coding and archetypal integration (9). Barbieri's *Code Biology^2^* describes cue–program relationships in which environmental signals activate biological programs through arbitrary yet conserved codes, including the principle that identical cues may map onto divergent responses depending on contextual rules (18, 19, 35). This logic parallels our account of archetypal coding, in which latent neural scripts are selectively released only when drive and cultural framing converge to lower threshold Φ. Therefore, ARCH × Φ advances Code Biology by formalizing this process within a scalar–vector equation, as a computational grammar of behavior. This formalism is consistent with classic ethological findings, such as Lorenz's imprinting and attachment theory, in which conserved scripts are gated by timing and context (36, 37). We now also ask if rigid beliefs and overvalued fixations—such as those observed in anorexia nervosa—might represent cultural/developmental "imprints," where early exposure and emotional salience tagging lower Φ and lock archetypal scripts into maladaptive trajectories?

### 1.1 Constraint-convergent execution

While ARCH × Φ was developed to model human behavior, its core logic—execution through the convergence of independent constraints within a threshold field as discussed by Buzsáki (38), appears across biological domains. In the canonical rodent lordosis model, estrogen priming (Drive), intact neural circuitry (Archetype), and temporal context (Cue, e.g., the presence of a male conspecific) must converge for the posture to be released by a central neural circuit (20). If any of these constraints are missing, the behavior collapses to zero.^3^ We previously applied this convergence logic to threat assessment, including mass shootings (9).

In the sections that follow, we will detail the neuroethological foundations of the ARCH × Φ model, define ten provisional archetypal systems, and demonstrate how scalar dynamics and threshold crossing organize behavior across clinical, forensic, and cultural contexts. We argue that behavior is neither random nor infinitely malleable—it is constrained by evolution, energized by affect, and shaped by meaning (3, 16). As we will discuss next, modern neuroscience views the brain not as a blank slate, but as a library of latent, evolutionarily shaped scripts that are continuously refined through learning and plasticity. These conserved grammars become behavioral events only when energized by Drive (D), selected by Culture (C), and released through threshold crossing (Φ).

## 2 Archetype neural scripts

Of the three terms in the ARCH equation, Archetype (A) represents the conserved neurocircuit substrate. Without it, behavior cannot emerge, regardless of the intensity of the drive or the cultural salience. We therefore begin our exposition with archetypes, the conserved neural scripts that provide the vectorial form of behavior. Drives and culture will be considered later as amplifiers and modulators, but the archetypal substrate must be established first. To operationalize the ARCH × Φ framework, we begin by detailing the neuroevolutionary foundations of *archetypal scripts*—the structured vectorial elements of behavior in this model. A close examination of the first term in the equation "A" reveals that inherited neural architectures scaffold behavior across species, comprising conserved motifs that organize perception, motivation, and action in response to evolutionarily salient contexts (3, 19–24).

The concept of the archetype was first articulated by Carl Jung (1959), who described these structures as innate, universal patterns of instinctual cognition and behavior.^4^ Notably, Jung emphasized their manifestation not only in myth and dream but also in animal behavior, anticipating later ethological insights (9, 10, 19), and described them as "guaranteed in every single individual" (10, 58).

The concept of archetypes as attractors originates with van Eenwyk, who applied chaos theory to analytical psychology, describing archetypes as strange attractors of the psyche—stable yet dynamic patterns around which thought, feeling, and behavior organize (28, 29). In dynamical systems, an attractor refers to the state or set of states toward which a system tends to evolve over time. A pendulum, for example, always settles at its lowest point, while the heart tends to beat in rhythmic cycles. Strange attractors are more complex: the system never repeats exactly, but its behavior is still constrained to a recognizable pattern, such as the unpredictable swirls of weather or turbulence. In psychology, van Eenwyk suggested that archetypes function in this way. Experiences of injustice may take many different forms, but they often orbit around the recurring archetypal motif of the victim seeking redress. The details differ, but the underlying attractor pattern remains recognizable. This view anticipates contemporary neuroscience, where surprise or prediction error can destabilize a system and shift it toward a new attractor state, much as unexpected events can activate latent archetypal scripts. Van Eenwyk later elaborated this framework, grounding archetypal dynamics in nonlinear systems theory (29). This was subsequently integrated with the attractor perspective, as outlined in Code Biology (18, 19), linking archetypes to biosemiotic coding systems. Vedor advanced this lineage with a psychobiological tripartite model, and more recently extended it into dream semiosis, framing dreams as code-based attractor processes (30, 31). Together, this work establishes an intellectual lineage in which archetypes are understood not as metaphoric abstractions but as conserved biological patterns with symbolic expression, constrained by attractor dynamics (28–35). The ARCH × Φ model builds directly on this lineage, extending it into neuroscience by specifying archetypes as structured vectorial scripts, operationally defined and testable within systems neurobiology and ethology.

Karl Pribram's notion of "neural programs for action" anticipated aspects of this view, describing behavior as organized by biologically grounded systems that integrate cortical and subcortical activity to guide goal-directed actions. Although not framed in culturally encoded symbolic cues, his model resonates with the idea that conserved neural circuits instantiate structured behavioral patterns—an intuition that ARCH × Φ formalizes within a computational grammar of archetypes (25). The recursive interplay between biological and symbolic coding ensures that behavior is neither reducible to neurochemistry nor infinitely malleable through culture but emerges from convergent coding across domains.

The convergence logic of ARCH echoes not only behavioral motifs but also other models of evolutionarily conserved neuronal circuits and cellular processes. For example, pupillary dilation has been modeled using deep learning and dynamical systems approaches, where the convergence of neural architecture (Edinger–Westphal circuit), arousal drive, and contextual luminance jointly determine the response. Detailed computational models that decouple these factors have shown how psychological state and luminance interact to shape pupil size, reinforcing the conjunctive logic of ARCH × Φ (39, 40). A similar biological code of convergence is observed in cellular processes, such as DNA replication, which requires multiple gating conditions to align before initiation (41). Within the ARCH framework, archetypes are further defined as vectorial scripts—structured patterns of affectively valenced behavior that specify the direction and structure of action. Their activation is neither automatic nor universal. Instead, it is conditional on scalar modulations: motivational intensity (Drive, D), symbolic–cultural reinforcement (C), and a threshold-based activation field (Φ). Behavior results only when these scalar factors converge sufficiently to release the latent script into execution (6, 9, 20). In this formulation:


$$Behavior=\vec{A}\cdot D\cdot C\cdot \Phi$$

These archetypal vectors are probabilistic rather than deterministic; humans are not innately afraid of snakes or heights per se, but they are biologically predisposed to acquire such fears more readily than they are culturally neutral stimuli—a phenomenon known as *prepared learning* (21). As Sapolsky notes, ethology's enduring insight is that evolution does not hard-wire specific behaviors but lowers the *learning thresholds* for context-sensitive adaptations (42). This principle is fundamental to the ARCH model: archetypes represent modular behavioral potentials that can be activated, inhibited, or rechanneled depending on context. Whereas prior work has largely remained descriptive, ARCH × Φ introduces a computational grammar of behavior that links archetypal activation to measurable circuits, scalar modulation, and threshold-dependent release. This framework thus bridges biosemiotics, affective neuroscience, and psychiatry, providing an integrative and testable model.

Our recent work in behavioral threat assessment has further supported this view (9). We argue that archetypes should be conceptualized as biologically grounded behavioral universals—modular, affectively charged motifs instantiated in conserved neural networks. These patterns are not static but developmentally tuned, culturally refracted, and symbolically elaborated. They display cross-cultural recurrence, emerge reliably in response to core ecological and social conditions, and possess identifiable neurobiological substrates. This reconceptualization strengthens the claim that archetypes are neurocognitive design constraints—latent action grammars sculpted by phylogenetic history and expressed through cultural variation.

We extend Jung and Barbieri's work by grounding archetypes in neural circuits—archetypal nervous systems—modular architectures specialized for recurrent adaptive problems such as defense, bonding, and competition (10, 18, 19, 42–45). In the ARCH × Φ framework, archetypal scripts are probabilistic information codes instantiated in cortico-limbic and subcortical pathways, where oscillatory synchrony and neurotransmitter dynamics shape salience (38, 45). These neural codes are nested within metacodes that link biological substrates to symbolic meaning (39). For example, caregiving is scaffolded by medial preoptic and oxytocinergic networks (15), but is culturally enacted as a healer, nurse, or parent. Archetypal vectors align perception, affect, motivation, and action; they can combine (e.g., Martyr + Avenger), inhibit one another (e.g., Caregiver suppressing Aggressor), or crystallize as *Fixed Archetypal Action Patterns^5^* (FAAPs) when symbolic cues amplify conserved scripts (9). Their orchestration—which we later formalize as Dynamic Archetypal Coordination (DAC)—underlies complex clinical, forensic, and cultural behaviors (see Section 5.1).

Building on this foundation, we now propose a structured taxonomy of ten canonical archetypal systems—the *Systema Behavorum*—which together define the vectorial architecture of ARCH × Φ. Having established archetypes as conserved neurocircuit substrates—vectorial templates that align perception, affect, motivation, and action—we now turn to their taxonomy. To make the ARCH × Φ framework operational, archetypes must be specified not only in abstract terms but also as identifiable neural systems with evolutionary continuity. We therefore propose a provisional set of ten canonical archetypes, the Systema Behavorum, which together constitute the foundational grammar of behavior. These systems provide the structured inputs on which drives and cultural modulation act, serving as the empirical anchor for testing the ARCH × Φ model.

## 3 Systema Behavorum

To formalize a new taxonomy, we define ten canonical archetypal neural systems, each corresponding to a distinct evolutionary domain of behavior and instantiated by conserved neurobehavioral circuits. These ten systems, collectively termed the *Systema Behavorum*, represent a provisional set of primary archetypes—a grammar of behavior rather than a definitive taxonomy. Each system is assigned both a symbolic label and a conventional descriptor, pairing evocative terminology with empirically grounded neurobiological constructs. The taxonomy should be understood as a structured input set for the computational grammar of ARCH × Φ, rather than as a closed or exhaustive classification.

It is essential to note that these ten systems are not definitive; they serve as a parsimonious starting point, informed by evidence from ethology, affective neuroscience, and psychiatry. Secondary and composite archetypes (e.g., Warrior, Martyr, Victim, Healer) emerge when these canonical systems are dynamically combined, culturally scaffolded, and symbolically elaborated (9). Additional motifs—such as victimhood or persecution—may therefore represent recurring composite archetypes that warrant further formalization in future iterations.

We define ten canonical archetypal neural systems, each corresponding to a distinct evolutionary domain of behavior and instantiated by conserved neurobehavioral circuits. These ten systems, collectively termed the *Systema Behavorum*, represent a provisional grammar of behavior rather than a definitive taxonomy. They are:

1. *Navigia*—goal-seeking and exploration
2. *Theromata*—caregiving and social bonding
3. *Phobon*—threat detection and defense
4. *Agonix*—competition and status striving
5. *Venex*—mating, sexual signaling, and reproductive behavior
6. *Sacrifex*—self-transcendence and symbolic devotion
7. *Thumos*—recognition, honor, and moral striving
8. *Imitati*—imitative learning and cultural acquisition
9. *Hedonix*—pleasure, play, and reward
10. *Alligantia*—joining, uniting, and developing coalitions

These systems comprise the foundational vector set of the ARCH × Φ model. While their form is biologically constrained, their expression is modulated by motivational drives, symbolic cues, and culturally conditioned thresholds. Each of these ten systems has empirically validated analogues in animal models. For example, rodent maternal behavior underlies Theromata, predator avoidance and freezing responses illustrate Phobon, dominance hierarchies in primates exemplify Agonix, song learning in zebra finches models Imitati, and rough-and-tumble play in rats (50 kHz vocalizations) demonstrates Hedonix. These animal analogues are elaborated within the system descriptions that follow, underscoring that the *Systema Behavorum* is grounded in conserved ethological motifs. Together, the *Systema Behavorum* constitutes a neuroethological grammar of action—a repertoire of archetypal templates capable of extensive recombination across development, context, and culture. We believe that these systems reflect recurrent adaptive problems as previously documented in ethology, affective neuroscience, and evolutionary psychology. **Table 1** summarizes the ten systems, highlighting their evolutionary function, symbolic expression, and clinical relevance. In the following section, we define and characterize these systems in detail, with a focus on their evolutionary function, neurobiological substrates, symbolic expression, and relevance to clinical psychiatry and social behavior.

**Table 1:** *Systema Behavorum*: canonical archetypal systems in the ARCH × Φ model.

System	Core function	Primary neural substrates	Clinical relevance
Navigia (Exploration/Goal-Seeking)	Exploration, novelty seeking, purposive navigation	Hippocampus (spatial mapping), dorsal striatum, VTA → NAc dopaminergic projections^45^	Restlessness, ADHD, compulsive novelty-seeking
Theromata (Caregiving/Bonding)	Parental care, nurturance, affiliative bonding	mPOA, ACC, NAc, oxytocin/vasopressin pathways^15^	Attachment pathology, caregiver burnout, enmeshment
Phobon (Threat Detection/Defense)	Fear, vigilance, defensive withdrawal or aggression	Amygdala (BLA/CeA), hypothalamus, PAG, HPA axis^48^	Anxiety disorders, PTSD, paranoia, hypervigilance
Agonix (Competition/Status Striving)	Rivalry, dominance assertion, status negotiation	VTA → NAc salience circuits, OFC–striatal loops, dlPFC; testosterone-modulated pathways^42^	Narcissistic pathology, chronic rivalry, power abuse
Venex (Mating/Sexual Signaling)	Courtship, libido, reproductive strategy	mPOA, medial amygdala, VTA → NAc reward circuits, HPG axis^55^	Hypersexuality, sexual inhibition, intimacy distortions
Sacrifex (Self-Transcendence/Altruism)	Altruistic devotion, prosocial sacrifice	mPFC, TPJ, DMN; serotonergic/oxytocinergic modulation^59^	Martyrdom, zealotry, ideology-fused suicidality
Thumos (Recognition/Moral Assertion)	Honor, moral striving, grievance-driven action	Anterior insula, mPFC, ACC, ventral striatum; serotonergic tone^59^	Moral rigidity, revenge ideation, grievance pathology
Imitati (Imitative Learning/Cultural Copying)	Observational learning, role adoption, mimicry	Mirror neuron system (premotor cortex, IPL), STS, mPFC; mesolimbic reinforcement^68^	Conformity, contagion, cultic mimicry, social-learning deficits
Hedonix (Pleasure/Reward-Seeking)	Reward-seeking, play, affective comfort	VTA → NAc reward circuits, endogenous opioids, vmPFC regulation^14^	Addiction, anhedonia, compulsive hedonic looping
Alligantia (Coalition/Group Cohesion)	Alliance-building, in-group bonding, group defense	TPJ, mPFC, ventral striatum; oxytocin/vasopressin pathways^72^	Tribalism, radicalization, intergroup aggression

Mpfc, medial prefrontal cortex; ACC, anterior cingulate cortex; NAc, nucleus accumbens; VTA, ventral tegmental area; PAG, periaqueductal gray; HPA, hypothalamic–pituitary–adrenal axis; HPG, hypothalamic–pituitary–gonadal axis; OFC, orbitofrontal cortex; dlPFC, dorsolateral prefrontal cortex; DMN, default mode network; TPJ, temporoparietal junction; STS, superior temporal sulcus; IPL, inferior parietal lobule; vmPFC, ventromedial prefrontal cortex; BLA/CeA, basolateral/central amygdala.

Pathway designations (e.g., VTA → NAc) are provisional and highlight the primary nodes most consistently implicated; additional connections are likely to emerge as evidence accumulates (e.g., animal, human, or mixed evidence).

The ten systems presented here were derived from converging evidence across three domains (1): conserved behavioral patterns documented in ethology and evolutionary psychology; (2) established neural systems associated with affective regulation, drive modulation, and social signaling; and (3) symbolic expressions of behavior that recur across cultural and historical contexts. Each archetype reflects a recurrent adaptive challenge, shaped by evolutionary pressures and retained in neural architecture. While these terms do not designate species, our proposed naming convention—*Systema Behavorum*—pays homage to Linnaean taxonomy in its attempt to classify foundational behavioral systems with precision and parsimony.

Although often associated with symbolic psychology, Jung also anticipated an ethological understanding of archetypes. He cited eel migration, wasp stinging, and bird navigation as examples of conserved behavioral templates unfolding through internal timing, instinctual drives, and environmental constraints (10). These observations parallel the later ethological work of Lorenz and Tinbergen (13, 22–24) and support the view that archetypes are evolutionarily grounded neural patterns or modules, activated through constraint-convergent mechanisms rather than conscious volition (28–35).

These ten systems are not exhaustive; rather, they represent our provisional foundational set, identified through phylogenetic conservation, cross-species continuity, and symbolic elaboration in humans, and are open to refinement as further empirical evidence accumulates. Computationally, Archetypal scripts (A) specify neuro-circuits; Drive (D) provides energetic amplification; Culture or Cue (C) supplies symbolic salience and metadata tags that bias script selection; and Threshold (Φ) gates whether weighted activations cross into expression. The resulting output is probabilistic, shaped by salience weighting and context. Dysregulation may occur through (a) excessive drive amplification, (b) cultural distortions such as supernormal stimuli that bias metadata tagging (see Section 9.2), or (c) threshold collapse, producing maladaptive or compulsive patterns.

### 3.1 Navigia system (exploration/goal-seeking)

The Navigia archetype derives from the Latin *navigare*, "to navigate," and encodes goal-directed exploration and purposive movement. Evolutionarily, it represents one of the oldest behavioral motifs, from chemotaxis in single-celled organisms to structured foraging and spatial navigation in vertebrates. As nervous systems evolved, this capacity developed into structured locomotion, foraging behavior, and spatial planning (44, 45).

In vertebrates, *Navigia* is subserved by hippocampal spatial mapping circuits, basal ganglia motor loops, and dopaminergic novelty-seeking pathways. In humans, it enables both literal navigation and abstract goal pursuit, such as academic planning or strategic decision-making. As a vector, *Navigia* is directionally aligned toward novelty, problem-solving, and adaptive foresight. It is typically activated under positive valence drives such as curiosity or mastery and suppressed under threat-dominant conditions. Neurobiologically, Navigia is mediated by hippocampal spatial mapping circuits, basal ganglia motor loops, and dopaminergic novelty-seeking pathways (45). In humans, Navigia governs both physical navigation and abstract goal pursuit. Dysregulation might contribute to restlessness, ADHD, or compulsive novelty-seeking.

### 3.2 Theromata (caregiving/bonding)

The Theromata archetype derives from the Greek *thermē*, "warmth," and encodes caregiving, affiliative bonding, and nurturance. Evolutionarily, it emerged in species that require extended parental investment, with expression evident in behaviors such as nest-building, nursing, grooming, and affiliative contact, observed across birds, mammals, and select aquatic taxa. In humans, Theromata is mediated by the medial preoptic area (mPOA), oxytocinergic and vasopressinergic pathways, and limbic-cortical regions such as the anterior cingulate and nucleus accumbens (46, 47). Rodent maternal behavior paradigms provide robust empirical evidence for this system: lesions or inactivation of the mPOA abolish pup retrieval, licking/grooming, and nursing, while the release of oxytocin and prolactin reliably facilitates caregiving behaviors (11, 15). Other animal studies further demonstrate that the distributions of oxytocin and vasopressin receptors predict pair-bonding, with receptor antagonists blocking affiliation and agonists enhancing partner preference (11). These converging findings underscore that Theromata is one of the most deeply conserved and experimentally validated archetypal systems. These circuits may underlie both instinctive parental responses and culturally elaborated caregiving roles, such as those of a teacher, therapist, or healer. As a behavioral vector, Theromata organizes proximity-seeking, protection, and empathic attunement. Dysregulation may contribute to attachment pathology, relationship control issues, or burnout in caregiving professions.

### 3.3 Phobon (threat detection/defense)

The Phobon archetype derives from the Greek *phobos*, "fear," and encodes threat detection, defensive action, and boundary enforcement. It is one of the most conserved behavioral systems, present even in simple organisms through the release of toxins or aversive motility, and further elaborated in vertebrates as coordinated defensive strategies. Among vertebrates, this system is instantiated in hypothalamic–periaqueductal gray (PAG)–amygdala circuits, which coordinate defensive aggression, withdrawal, or immobilization (48, 49).

In humans, *Phobon* underlies behaviors ranging from physical self-defense to ideologically framed perception of threat. It is often recruited in hypervigilant states or grievance-laden worldviews, where symbolic threats (9, 42)—such as ideological outgroups—are perceived as existential dangers. Vectorially, it directs attention and action toward avoidance, vigilance, and perimeter control. *Phobon* becomes clinically relevant in anxiety disorders, paranoia, or radicalized threat schemas.

### 3.4 Agonix (competition/status striving)

The Agonix archetype derives from the Greek *agon*, meaning "contest" or "struggle," and organizes behaviors centered on competition, status negotiation, and dominance assertion. It is evolutionarily conserved and expressed across species: insects utilize vibratory or acoustic displays, amphibians and reptiles engage in ritualized combat, and primates navigate social hierarchies through alliances, grooming, and dominance posturing.

Neurobiologically, *Agonix* is supported by mesolimbic dopaminergic circuits that mediate motivational salience and reward pursuit, as well as orbitofrontal regulatory systems involved in social evaluation and decision-making. Additionally, prefrontal-striatal loops subserve performance monitoring and inhibitory control (39, 49–52). In humans, this vector structures ambition, rivalry, achievement striving, and leadership dynamics, and is oriented toward upward social mobility and the acquisition of prestige (9).

When dysregulated—particularly under conditions of identity fusion, chronic narcissistic reinforcement, or impaired social feedback—*Agonix* may underlie psychopathological states such as narcissistic personality disorder, grandiose self-schema, dominance ideation, or institutional power abuse (42, 43, 53). Symbolically, the rattlesnake offers an ethological metaphor for *Agonix*: its threat display conserves energy while asserting territory, reflecting the archetype's strategic calibration between deterrence and aggression.

At the prosocial and collective level, *Agonix* informs structured competition (e.g., politics, law, economics). However, in destructive extremes, it manifests in predatory behaviors such as sexual exploitation, coercive resource acquisition, and ideological conquest. War—ritualized, legitimized, and often glorified—may be understood as a macro-social expression of *Agonix*, wherein dominance, control, and prestige are pursued under collective banners (see **Table 2**). The acquisition of nuclear weapons represents its apex: a symbolic assertion of supremacy and deterrence that, when activated, becomes a weapon of catastrophic destruction.

**Table 2:** Coordinated activation of archetypal systems during intergroup conflict.

Archetypal system	Expression in tribal conflict
Phobon (Defense)	Threat vigilance, fight-or-flight readiness
Agonix (Competition)	Status assertion, combativeness
Thumos (Recognition)	Honor defense, retaliation rituals
Alligantia (Coalition)	Group bonding, alliance loyalty
Imitati (Mimesis)	Adoption of enemy/friend group symbols, war dances
Sacrifex (Devotion)	Martyrdom, ideological self-sacrifice
Venex (Signaling)	Sexual dominance displays, post-victory rituals

### 3.5 Venex (mating/sexual signaling)

The Venex archetype derives from the Latin venere, "to love" or "to sexually engage," and governs mating behavior, sexual signaling, courtship, and reproductive strategy. It is among the most ancient motivational systems, conserved across all sexually reproducing species. Venex integrates both biological imperatives—libido, pair bonding, reproductive drive—and culturally elaborated expressions of sexual identity, ritual, and display. *Venex* is rooted in hypothalamic–pituitary–gonadal (HPG) axis regulation, with testosterone, estrogen, and oxytocin modulating mating interest and attachment. Neural substrates include the ventral tegmental area (VTA), medial preoptic area (mPOA), amygdala, and limbic reward systems (42, 54, 55). In humans, *Venex* is elaborated through symbolic roles (e.g., seducer, romantic, parenthood aspirant) and can intersect with social norms, identity formation, and moral codes. Freud's early theories of libido anticipated this dual structure, linking instinctual drives to culturally constrained expression. Vectorially, *Venex* orients behavior toward sexual signaling, courtship rituals, and pair bonding. Dysregulation may manifest as hypersexuality, sexual inhibition, compulsive pursuit of validation, or distortions in relational intimacy (56, 57). Culturally, it is often modulated through ideologies of purity, shame, reproduction, or desire.

### 3.6 Sacrifex (self-transcendence/altruism)

The Sacrifex archetype derives from the Latin *sacrificium*, "sacred act," and encodes symbolic devotion, self-transcendence, and altruistic offering. Evolutionarily, it supports group cohesion, kin altruism, and prosocial commitment, with parallels in eusocial insects, cooperative mammals, and human ritual practice (42, 58).

In humans, *Sacrifex* is instantiated in medial prefrontal (mPFC) and temporoparietal junction (TPJ) regions, associated with identity fusion, moral cognition, and spiritual awe (59–61). It is modulated by serotonergic and oxytocinergic signaling. *Sacrifex* is often expressed in acts of charity, ritual abstinence, martyrdom, or legacy-seeking. Functionally, Sacrifex modifies behavioral thresholds by assigning disproportionate salience to self-directed cost when framed as group-beneficial or morally significant. Neurobiologically, mPFC–TPJ coupling integrates social perspective-taking with value assignment, while serotonergic tone modulates inhibition of self-preservation drives. When cultural coding elevates Sacrifex salience, Φ is lowered, making costly prosocial actions more probable. This process can be formalized as a suppression of survival-oriented drive signals, where Sacrifex salience tags override default cost-avoidance metadata, reallocating motivational energy toward group-aligned outputs. In adaptive states, this supports altruism and cohesion; in pathological states, threshold dysregulation produces rigid missionality or ideology-fused suicidality (9, 62, 63).

### 3.7 Thumos (recognition/moral assertion)

The Thumos archetype, derived from the Greek *thymos* ("spiritedness," "moral striving"), encodes the drive for recognition, dignity, and honor-based moral assertion (9). Evolutionarily, it is related to status regulation but diverges from Agonix in its ethical and existential orientation: whereas Agonix seeks competitive dominance, Thumos defends reputation, justice, and symbolic legacy (9, 42). Functionally, it integrates emotional memory, grievance tracking, and dignity restoration (59).

Neuroanatomically, Thumos engages the anterior insula, medial prefrontal cortex (mPFC), anterior cingulate cortex (ACC), and ventral striatal systems, interacting with monoaminergic regulation (9, 51, 64). Within the ARCH × Φ framework, it operates as a recognition-sensitive threshold regulator: humiliation or perceived injustice elevates motivational drive (D), while cultural coding amplifies symbolic salience (C), jointly lowering Φ for honor-restoring scripts (9, 59). ACC–mPFC–ventral striatal–insula networks assign high weight to grievance-linked signals, biasing selection toward dignity-restoring behaviors (9, 51, 64).

Culturally, Thumos animates hero narratives, principled protest, redemptive violence, and symbolic status reclamation. Balanced regulation supports moral courage and principled leadership. Dysregulation arises when grievance salience is overvalued, suppressing affiliative or inhibitory archetypes, and leading to righteous retribution, targeted attacks, or ideologically motivated suicide (9, 59–66).

In forensic contexts, thymotic drive is frequently implicated in extreme overvalued beliefs (EOBs) and ideologically motivated violence, where symbolic grievance and recognition motives converge to energize otherwise latent archetypal scripts (9, 62, 63).

### 3.8 Imitati (imitative learning/cultural copying)

The Imitati archetype derives from the Latin *imitatio*, "to imitate," and governs observational learning, behavioral mimicry, and symbolic role adoption. It facilitates the internalization of social scripts through exposure to exemplars, enabling rapid knowledge transmission and alignment with group norms. Evolutionarily, Imitati supports social cohesion and adaptive efficiency, particularly in young or subordinate individuals acquiring context-sensitive behavior from peers or prestige models. In primates, imitation contributes to tool learning, alliance formation, and the rehearsal of complex social sequences (42, 43, 67). Across species, coordinated action—such as marching, shoaling, grooming rituals, or collective displays—often arises from entrained mimetic circuits that enable organisms to move, act, or respond in temporal synchrony with conspecifics (42, 43, 67).

Neurobiologically, *Imitati* is anchored in the mirror neuron system, particularly in the premotor cortex and inferior parietal lobule, which supports the simulation and rehearsal of observed actions. The medial prefrontal cortex and superior temporal sulcus contribute to social tracking and model selection, while mesolimbic reward pathways reinforce successful mimicry and group alignment (68, 69). In symbolic systems, *Imitati* enables the acquisition of ritual, ideology, and social identity through representational copying. Dysregulation may underlie conformity, mimicry-based contagion, or developmental deficits in social learning.

### 3.9 Hedonix (pleasure/reward-seeking)

The Hedonix archetype derives from the Greek *hēdonē*, "pleasure," and governs behaviors of pleasure-seeking, affective comfort, and reward reinforcement. Evolutionarily, it functions to strengthen adaptive states such as feeding, rest, grooming, and play by pairing them with affective gratification. Across species, self-soothing and rhythmic behaviors reflect the operation of this system in regulating arousal and promoting homeostasis. In rodents and primates, for example, tickling induces vocalizations associated with positive affect and social approach (70), suggesting that pleasure, such as playing and tickling, has a conserved neural basis (71).

Neurobiologically, *Hedonix* is mediated by mesolimbic dopamine circuits, endogenous opioids, and regulatory feedback from the ventromedial prefrontal cortex (14, 42, 51). These systems track affective salience and help gate the behavioral availability of comfort-seeking scripts. Activation may be reflexive (e.g., touch, warmth) or symbolically evoked through culturally conditioned rituals. In symbolic expression, *Hedonix* underlies behaviors such as feasting, music, recreational play, and spiritual euphoria.

When dysregulated, however, the Hedonix system becomes vulnerable to hijacking by exogenous agents (e.g., opioids, cocaine) or symbolic amplifiers (e.g., video games, pornography). Opioid addiction exemplifies pharmacological hijacking, wherein sustained elevation of μ-opioid tone bypasses natural gating thresholds, narrowing symbolic repertoires around drug-seeking behavior (14, 50, 70). Video game addiction represents a parallel form of symbolic drive amplification, in which immersive, feedback-rich environments engage Hedonix and Thumos via synthetic reward hierarchies and status simulacrum (26, 42). In both cases, the collapse of regulatory thresholds (↓Φ) leads to compulsive behavioral looping, affective flattening outside the addictive context, and progressive erosion of naturally scaffolded symbolic engagement (14, 42).

### 3.10 Alligantia (coalition/group cohesion)

The Alligantia archetype derives from the Latin *alligare*, "to bind," and governs coalition formation, group cohesion, and symbolic affiliation. Ethological evidence for this system is found in coordinated alliances among chimpanzees, strategic grooming-based hierarchies, nest defense in eusocial insects such as ants and bees, and collective defense behaviors across primates and other social mammals (42, 43, 67, 72). In humans, it supports factional identity, ideological alignment, and symbolic group rituals—often in conjunction with *Imitati*—including oath-taking, chanting, and uniformed display. When dysregulated or culturally amplified, this system may contribute to large-scale intergroup aggression, ideological extremism, and terrorism.

Neurobiologically, *Alligantia* is supported by the temporoparietal junction (TPJ) and medial prefrontal cortex (mPFC), structures involved in social perspective-taking and trust valuation (51). The striatum reinforces cooperation, while oxytocin and vasopressin enhance in-group bonding and loyalty under threat (72). Reciprocal modeling and shared enemy detection often gate the expression of this system. In symbolic form*, Alligantia* underlies nationalism, tribalism, groupthink, and identity fusion. It interacts with *Thumos* (recognition), *Agonix* (competition), and *Phobon* (defensive mobilization), and may be potentiated by collective stress or perceived marginalization. Dysregulation might lead to exclusionary moralism, radicalization, or intergroup aggression (9, 62, 63). In computational terms, Alligantia assigns elevated salience weights to in-group metadata tags, lowering Φ for coalition-concordant behaviors while simultaneously raising thresholds for affiliative scripts directed at out-groups. **Table 1** provides a consolidated overview of these ten systems, highlighting their evolutionary function, neurobiological substrates, symbolic expression, and clinical relevance.

### 3.11 Clarification on scope and interpretation

While we have defined ten canonical archetypal systems, several clarifications are necessary. First, the ten systems are intended as primary, phylogenetically conserved archetypes, whereas secondary or composite archetypes—such as Warrior, Martyr, Healer, or Victim—are culturally elaborated roles built upon these primary systems. Second, symbolic culture provides the scaffolding through which conserved systems are expressed as recognizable identities. For example, victimhood may emerge from the convergence of Phobon (threat detection), Thumos (recognition and grievance), Sacrifex (self-transcendence), and Alligantia (coalition affiliation), stabilized by cultural narratives of injury and injustice. Third, although the primary set is finite, their possible recombinations are vast, especially when modulated by Drive (D), Culture (C), and Threshold (Φ). The grammar is bounded, but the symbolic repertoire is functionally unbounded. For example, caregiving (Theromata) may evolve into the cultural role of healer or saint; threat-defense (Phobon) may develop into a warrior or avenger; and sacrificial devotion (Sacrifex) may expand into martyrdom. Finally, this taxonomy is explicitly provisional. It is offered as a parsimonious starting point, open to refinement, stratification, or expansion as further empirical data accumulate.

### 3.12 Functional necessity of archetypal integrity

A central prediction of the ARCH × Φ model is that behavior will collapse if the underlying archetypal substrate (A) is nonfunctional, regardless of how strong the scalar amplifiers (Drive, Culture, or Φ) may be. This has been empirically tested across multiple systems. For example, lesions to the medial preoptic area (mPOA) eliminate maternal caregiving (*Theromata*), amygdala damage suppresses fear responses (*Phobon*), and hippocampal or dopaminergic disruption impairs exploration (*Navigia*). Competitive behaviors diminish with orbitofrontal or striatal lesions (*Agonix*), while damage to the ventrolateral subdivision of the ventromedial hypothalamus (VMHvl) abolishes lordosis in hormonally primed females (Venex). Likewise, lesions to the anterior cingulate cortex (ACC) or temporoparietal junction (TPJ) reduce prosocial helping (*Sacrifex*), and ventromedial prefrontal cortex (vmPFC) injury flattens grievance-driven behavior (*Thumos*) (15, 42, 46, 48, 51).

Damage to mirror neurons networks can inhibit learning (68, 69). These findings reinforce the ARCH principle: when A ≈ 0, the behavioral product Φ × (A × D × C) approaches zero. Archetypes in our construct are not metaphors—they are conserved, embodied neural programs whose presence is a necessary condition for the emergence of structured behavior.

The ten preliminary systems define the vectorial structure of the ARCH equation. Their activation requires scalar convergence across motivational energy (*Drive*), symbolic-cultural reinforcement (*Culture*), and threshold readiness (*Φ*). By formalizing this taxonomy, the ARCH × Φ framework provides a clear and actionable lens for decoding behavior that is structured, symbolic, and biologically intelligible. Together, these ten systems establish the structural substrate of the ARCH equation (A). In the sections that follow, we examine how their activation depends on the energetic amplifiers of Drive (D), symbolic modulation by Culture (C), and context-sensitive gating through Threshold (Φ). Next, we examine how these systems interact—sometimes competitively, sometimes synergistically—through the process of Dynamic Archetypal Coordination.

## 4 Stratified archetypes

Having established that each primary archetype is instantiated in conserved neural architecture—and that lesion studies across species validate their necessity for behavioral expression—we now examine how these scripts are further elaborated through evolutionary and cultural scaffolding. While the presence of the archetypal substrate is a necessary foundation, the behavioral expression of these archetypes is not fixed; rather, it evolves through stratified layering of symbolic meaning, developmental timing, and social reinforcement. This concept of stratified layering integrates insights from ethology, neurodevelopment, and cultural psychology. While Jung, Lorenz, Hoffmeyer and Sapolsky theorized that higher systems elaborate or inhibit more primitive responses, ARCH × Φ extends this tradition by formalizing stratification as a computational grammar: primary archetypes represent conserved neural scripts. In contrast, secondary archetypes emerge as culturally scaffolded variants within the same vectorial framework. Echoing the principle of hierarchical dissolution and symbolic mediation, ARCH proposes that behavior emerges through the progressive elaboration of ancient neural motifs by newer, culturally encoded schemas (42, 73–76). Evolution appears to favor a stratified structure for these behavioral templates, wherein primary archetypes are phylogenetically conserved neural scripts (e.g., caregiving, aggression, status-seeking), while secondary archetypes represent culturally elaborated variants scaffolded atop these ancestral circuits. This scaffolding mirrors the increasing complexity of nervous systems: simple organisms operate via hardcoded archetypes (e.g., foraging, escape), whereas mammals and humans construct increasingly symbol-laden scripts (e.g., *Warrior → Soldier → Martyr → Suicide Bomber*) (9, 62, 63). Thus, archetype neural scripts likely evolve by phylogenetic scaffolding (9). The ARCH × Φ framework is teleonomic: archetypal systems are purposive in the sense that they reliably orient behavior toward adaptive ends. Archetypal activation is stochastic, probabilistic, and context-dependent, governed by convergent scalar–vector dynamics. This prevents deterministic misinterpretations while preserving the evolutionary logic of purposive, yet non-teleological, action.

As another example, the Caregiving archetype is observed in fish and birds through parental defense behaviors (23). The hierarchical modulation of such behaviors is now evident in modern neurological insights, which show that higher systems evolve to inhibit and refine more primitive responses—a principle foundational to the layered architecture of the ARCH model. In primates, caregiving is expanded by empathic circuitry; in humans, it becomes symbolically codified through culturally prescribed nurturing roles and sacrificial ideologies (42, 43, 67). Where Lorenz described fixed action patterns as instinctive motor programs (22, 23), composite archetypes represent higher order recombinations of such modules, shaped by cultural learning and cortical oversight (27, 32, 42). Behavior thus evolves not as a linear sequence, but through the elaboration and stacking of archetypal layers. This is similar to von Uexküll's notion of the Umwelt (1934) (75), in which each organism experiences the world through evolutionarily shaped perceptual and motivational filters (74–76).

## 5 Integrative archetypes

This ethological model exemplifies how archetypal motifs are both biologically grounded and contextually gated—precisely the convergence logic formalized in ARCH × Φ. Recent neuroimaging work confirms this view: fMRI studies in awake newborn chicks show that imprinting memory engages distributed associative and higher-order regions, demonstrating that conserved attachment scripts can be localized and tracked at the neural-systems level (77). This adds a modern neuroscience perspective to Lorenzian imprinting (36), which has served as a model for memory formation and attachment theory^6^ for decades (37), and highlights how experience-dependent convergence produces lasting neural change—a logic consistent with ARCH × Φ.

Seemingly complex human roles often emerge through the synthesis of multiple archetypal neural systems into unified behavioral identities. These composite archetypes reflect coordinated activation of evolutionarily conserved modules, shaped by cultural norms and emotionally salient contexts. Consider the physician: the Theromata System (Caregiver archetype) governs nurturance and affiliative behavior, rooted in mammalian parental care. Coupled with the Sacrifex System (Healer archetype), it supports moral commitment, empathic attunement, and the willingness to bear others' suffering (45). The Navigia System (Craftsman archetype) adds goal-directed mastery and procedural precision, as seen in primate tool use and human apprenticeships. Emotional amplifiers—such as awe, which enhances salience and lowers thresholds for Sacrifex and Thumos (61)—further potentiate these integrations, infusing technical practice with symbolic devotion.

Such enactments do not require new neural architecture but reflect the dynamic orchestration of existing scripts. Composite archetypes like the physician illustrate how the brain integrates multiple systems to produce behavior that is cognitively flexible, emotionally resonant, and evolutionarily grounded. This synthesis satisfies Tinbergen's four levels of explanation: it develops ontogenetically through learning and mentorship; it is mediated by substrates such as caregiving networks and dopaminergic tone; it reflects phylogenetic continuity with social mammals and primates; and it functions adaptively by sustaining prosocial, identity-defining roles (24, 43, 78). In ARCH × Φ terms, composite roles emerge from the convergence of internal forces (A, D, Φ) with external cultural and symbolic framing (C). As Sapolsky notes, evolution does not hard-wire behavior but tunes the thresholds and sensitivities of behavioral systems—like adjusting the dials of a radio rather than flipping an on/off switch (42). This captures the graded, context-sensitive, and probabilistic nature of ARCH × Φ.

### 5.1 Formalizing composite activation: dynamic archetypal coordination

At any given moment, multiple behavioral scripts compete for expression. The decision to eat, go to work, or rest does not arise from a single cause but from the interaction of several archetypal systems. A hunger drive may activate Navigia (exploration) and Hedonix (reward-seeking), while obligations and goals recruit Agonix (competition/status) and Thumos (recognition). Fatigue, in turn, may raise the threshold for all but Hedonix (comfort, rest). Which script prevails depends on three factors: its relative salience in context, whether its threshold for activation is low enough to cross into behavior, and how it is influenced by other scripts that may amplify or suppress it.

We refer to this dynamic interplay as Dynamic Archetypal Coordination (DAC)—the process by which multiple conserved systems are orchestrated into coherent action. DAC extends the ARCH × Φ framework from single-script activation to the real-time coordination of multiple scripts. In everyday life, DAC explains why choices feel like weighing options, but in fact reflect the neural arbitration of archetypal systems competing for expression. These same dynamics scale upward into more elaborate contexts. In caregiving professions, Theromata (care) may be amplified by Sacrifex (self-transcendence), producing devotion that extends beyond instinct. In terms of ideological commitment, Thumos (recognition), Alligantia (coalition), and Sacrifex may align to sustain loyalty and promote self-sacrifice. In pathological states, DAC may become distorted—for example, when Phobon (threat) dominates and suppresses affiliative scripts, or when Hedonix (pleasure) loops compulsively under addictive conditions.

Neurobiologically, DAC corresponds to the shifting of connectivity among conserved circuits, as hormones, neuromodulators, symbolic cues, and cultural context modulate them. Evolutionarily, it reflects a multi-level synthesis: ancient scripts integrating in real-time with cultural overlays to produce adaptive—or maladaptive—actions. Up to this point, we have detailed the structural dimension of behavior—archetypes as conserved neurocircuit substrates, their stratification into symbolic roles, and their dynamic coordination into composite enactments. However, next structure alone is inert. For latent scripts to cross threshold Φ, they require motivational energy. This scalar amplification is provided by Drive (D), the second term of the ARCH equation. In the next section, we examine how drives—from hunger and pain avoidance to recognition and sexual desire—energize archetypal templates, modulate their salience, and, when dysregulated, distort their coordination into maladaptive forms.

## 6 Drive: the motivational engine of archetype activation

In the ARCH × Φ framework, Drive (D) functions as a scalar amplifier: it does not determine the form of behavior—which is encoded in Archetype (A)—but modulates its intensity, activation probability, and trajectory. Operationally, D indexes the motivational energy available to latent archetypal scripts as they approach threshold (Φ). Drives span a continuum from homeostatic imperatives (e.g., hunger, pain avoidance) to complex affective and symbolic motivators (e.g., recognition, status, sexual desire, nurturance). Biologically, these states are instantiated in subcortical circuits and modulated by neuromodulators, including dopamine, serotonin, oxytocin, testosterone, and cortisol (11, 42, 79–82). Neurosteroids, including allopregnanolone and DHEA, further calibrate excitatory–inhibitory balance by altering receptor sensitivity and GABAergic tone, thereby tuning archetypal salience weights across development (82–84). They thus represent a molecular bridge linking endocrine state, circuitry function, neural plasticity, and the probabilistic expression of conserved behavioral grammars. In this way, Drive is a measurable, mutable parameter within the computational grammar of behavior, linking endocrine state, circuit function, and symbolic readiness into a probabilistic model of action.

### 6.1 Scalar amplification and dynamic archetypal coordination dysregulation

When drive is elevated, even modest stimuli can activate multiple archetypes simultaneously. For example, a high-performing college student under pressure may experience coactivation of the *Agonix* system (competition), *Theromata* (self-directed care), and *Sacrifex* (neglect of physical needs in pursuit of ideals). This is not disorganized behavior but a coordinated distortion of DAC, where amplified drive and symbolic salience push the system into maladaptive overexpression. Mania (via kindling mechanisms) or amphetamine intoxication exemplifies states of endogenous or exogenous drive amplification, where elevated neuromodulatory tone dramatically increases motivational drive (D). This can hyperactivate archetypal schemas such as *Agonix* (competition), *Thumos* (recognition), or *Hedonix* (pleasure-seeking), even under the absence of cultural elicitation. The result is a collapse of threshold regulation (↓Φ), manifesting as impulsivity, grandiosity, and disinhibited symbolic behavior.

Neurobiologically, states like chronic stress elevate cortisol, deplete neurosteroids and disrupt serotonergic tone among other changes, leading to attentional narrowing and affective dysregulation (82–84). These changes increase the salience weighting (wi) and reduce the behavioral threshold (Φ), thereby making specific scripts more likely to be activated. Drive thus functions as both amplifier and potential destabilizer.

### 6.2 Physiological substrates of drive

Key drives map to well-established biological systems. What is described below is a simplified descriptive version of highly complex neural circuitry:

- Hunger: mediated by ghrelin and hypothalamic arcuate nucleus (85); activates *Navigia* and *Phobon*.
•Sexual desire: regulated by hypothalamic-pituitary signaling and modulated by testosterone, estrogen, dopamine, and oxytocin (20, 50, 54, 86); activates *Venex* and *Theromata.*
- Pain avoidance: integrates nociceptive input and limbic patterning (87); can activate *Phobon* and *Agonix.*
- Social bonding: supported by oxytocinergic and serotonergic systems (15, 72); energizes *Theromata* and *Sacrifex.*
- Status/reward seeking: mediated by dopaminergic tone (51); modulates *Thumos* and *Agonix.*

These systems do not guarantee behavioral output. Instead, they modulate scalar Drive (D), altering the readiness probability of archetypes to cross threshold (Φ) into expression.

### 6.3 Drive depletion and scalar failure

Just as excessive drive can distort behavior, drive depletion can suppress it. Even when archetypal structure (A) and symbolic context (C) are intact, low D prevents script execution.

- Parkinson's disease (88, 89): Dopaminergic depletion reduces the Seeking drive, attenuating *Navigia, Agonix*, and *Thumos* expression. Behavior becomes slow, effortful, or fails to initiate despite intact archetypes and social roles.
- Estrogen suppression (e.g., breast cancer treatment (90)): Leads to blunted affiliative and sexual drive. Patients may report reduced emotional intimacy, libido, or motivation for caregiving, reflecting attenuation of *Venex* and *Theromata*.
- Orchiectomy/testosterone suppression (91): Dampens competitive and sexual drives. This may reduce *Agonix* and *Venex* activation even when cultural and relational cues are present.
- Depression: Often features global drive suppression (hypodopaminergic state), raising Φ across systems and reducing behavioral initiation (88, 92).

These cases illustrate that behavior may be biologically impossible when scalar energy is insufficient, regardless of symbolic relevance or neurocognitive intactness.

### 6.4 Theoretical integration

Drive (motivation) is a biological variable, not a metaphor. It is mediated by specific circuits in brain and modulated by hormonal states, neurochemical signals, and environmental conditions. Within ARCH, Drive helps to explain why some archetypes remain latent, others become dominant, and some shift across time.

Within ARCH × Φ, Drive is a scalar variable that can be measured, manipulated, and modeled. It links endocrine state, circuit dynamics, and symbolic salience into a unified computational framework. In this way, Drive becomes a testable parameter rather than a descriptive label, preserving biological specificity while clarifying clinical meaning. This approach avoids both mechanistic reductionism (93) and theoretical pluralism without integration.

## 7 Dynamic coordination of emotional drives

Emotional drives do not act in isolation. Affective behavior results from network-level integration of interactions among neuromodulators, limbic-cortical circuits, symbolic meaning systems, and temporal context. Emotional expression and behavioral regulation depend on the dynamic interplay between limbic drivers and cortical control systems, not on the presence or absence of a single chemical messenger (11, 48). For instance, serotonin, dopamine, and cortisol modulate salience attribution, goal direction, and inhibition; however, they do not independently determine behavior (94). Instead, it is the patterned convergence of drives with archetypal architecture (A), symbolic encoding (C), and threshold readiness (Φ) that determines output.

### 7.1 Functional role in the ARCH × Φ equation

Emotional drives serve as a subdomain within Drive (D) in the behavioral equation:


$$Behavior=\vec{A}\cdot D\cdot C\cdot \Phi$$

They illustrate why the same archetype (e.g., Agonix or Theromata) may remain latent in one context yet activate in another: Drive (D) operates as a variable scalar, incorporating emotional intensity and physiological state, which modulates activation probability in a context-sensitive manner.

## 8 Archetype neural module activation

Why does a goose automatically retrieve an egg when it rolls out of the nest, while a human might erupt in outrage at an insult posted online? In both cases, latent behavioral templates are mobilized by specific triggers, but in humans, these triggers can be symbolic, cultural, and moral rather than purely sensorimotor.

In the ARCH × Φ model, archetypal activation is not continuous but conditional, dependent on latent neural scripts being mobilized by appropriate internal and external triggers. These archetypal templates are neurobiologically scaffolded but symbolically primed, often through developmentally sensitive periods in which emotional learning, cultural imprinting, and social exposure shape which scripts become dominant or repressed.

This process builds on Lorenz and Tinbergen's foundational work on fixed action patterns (FAPs) and innate releasing mechanisms (13, 22, 23, 39). However, it extends their logic into symbolic, moral, and identity-based domains of human behavior (9, 40). Just as a goose retrieves a displaced egg when it sees it outside the nest, a human may activate an archetypal defense script not only in response to a direct threat, but also to symbolic grievances, such as betrayal, injustice, or humiliation. In the human cognitive environment, symbolic releasers serve a function analogous to innate releasing mechanisms in animals: they trigger latent neural scripts through culturally coded cues, such as language, imagery, ritual, or ideology. These releasers may become supernormal when exaggerated by social media, political myth, or identity-based narrative, intensifying the salience of the stimulus and lowering the threshold (Φ) for archetypal activation (9, 13, 23, 39, 40).

Moreover, many of these symbolic cues are imprinted during critical windows, when experiences of status, loyalty, salience, pain, purity or injury become neurally tagged and later reactivated under similar affective conditions (3, 42, 77, 94). In this way, symbolic imprinting embeds culturally saturated meanings into the activation logic of evolutionarily conserved behavior. This is consistent with Erikson's view that identity formation unfolds through stage-specific social challenges, during which symbolic and relational meanings become developmentally encoded and later behaviorally reactivated.

As Sapolsky notes (42), modern symbolic environments can hijack ancient neural systems, causing behaviors such as aggression, sacrifice, or moral outrage to be deployed in response to abstract or virtual cues far removed from their ancestral triggers (26, 63). States such as awe—evoked by ideological grandeur, moral purity, or collective ritual (61, 64)—might further lower threshold Φ and potentiate archetypes like Sacrifex or Thumos. ARCH models this dynamic as the convergence of archetypal structure (A), scalar drive (D), cultural encoding (C), and context-sensitive threshold modulation (Φ).

To capture this, we introduced Fixed Archetypal Action Patterns (FAAPs) (9): evolutionarily conserved behavioral schemas that are culturally encoded and context-sensitive, triggered not only by sensorimotor stimuli but also by symbolic meaning and narrative framing. A moral injury, threat to status, or ideologically charged affront—for example, the commitment of a "violent true believer" (95)—can act as a symbolic releaser, directing aggression toward targets such as schools or government buildings (60). Such triggers may initiate the Warrior archetypal script (9), which mimics phylogenetically older scripts also seen in chimpanzees, including defense (Phobon), protest (Thumos), or sacrifice (Sacrifex) (42, 43, 67). Importantly, FAAPs can remain dormant until a threshold (Φ) is crossed, whether through rising Drive (e.g., methamphetamine intoxication) or context-specific cues (e.g., supernormal stimuli, see Section 9.2). This dynamic allows behavior to appear sudden or "disproportionate," when in fact it reflects the nonlinear convergence of archetype (A), drive (D), culture (C), and threshold (Φ) over time. Drives supply the energetic force for archetypal activation, but they are never expressed in a vacuum. Human behavior is always embedded in symbolic and social contexts that assign meaning and direction. Hunger may become ritual fasting, fear may transform into paranoid suspicion, and sexual desire may manifest as romantic courtship or altruistic devotion. These transformations illustrate why the ARCH × Φ model includes Culture as its third core variable: not as a peripheral overlay, but as an active causal partner that amplifies, suppresses, and shapes the expression of conserved scripts.

## 9 Culture: the symbolic and social frame

Culture is the third core variable in the ARCH × Φ equation, and the most explicitly symbolic. It provides the learned, narrative infrastructure through which behavior is shaped, sanctioned, or suppressed. Whereas archetypes supply form, and drives supply energy, culture (C) governs direction, salience, and legitimacy. It is not simply context, it is causal. Culture encodes meaning through language, ritual, norm systems, and institutional frameworks. It influences which archetypes are reinforced, which are inhibited, and what forms they take. A society may valorize martyrdom, suppress eroticism, or reframe caregiving as heroic or sacrificial. Culture thus modulates behavior probabilistically: amplifying the expression of some scripts while attenuating others, often without altering the biological substrate. For example, as shown in the lordosis model, female mice with intact hormonal priming and neural circuits may fail to express sexual receptivity if exposed to adverse rearing conditions (20), illustrating how C (context and experience) can suppress behavior even when A and D are intact. In ARCH terms, culture is a multiplier of probability: it does not generate archetypes or drives, but it amplifies or inhibits their expression through framing, social learning, and meaning-making (9). This explains why behavior with a shared biological basis can appear radically different across contexts, roles, or civilizations (42, 43).

### 9.1 Culture as supernormal stimulus

In ethology, exaggerated versions of evolved cues (e.g., hyper-colored mates or oversized eggs) are well established to elicit stronger responses than the natural stimuli. They are referred to as "supernormal stimuli." ARCH extends this logic into the symbolic domain (13, 22, 36, 42). Evolutionary psychologists have described modern-day supernormal stimuli and documented how cultural artifacts, including fast food, pornography, and digital media, exploit evolved biases by overstimulating perceptual and motivational systems (42, 96). From an ARCH × Φ perspective, such inputs function as salience amplifiers, increasing the weighting (wi) of specific archetypal scripts within Dynamic Archetypal Coordination (DAC). Symbolic exaggerations act as metadata overlays, tagging certain cues with disproportionate informational value, which lowers Φ and shifts behavioral probability. In this way, supernormal stimuli do not simply 'hijack' circuits in a metaphorical sense; they alter the computational parameters of script selection and threshold regulation, producing coherent but maladaptive behavioral outputs (96). This likely reflects the primary emotion some would call "surprise"—an evolutionarily conserved mechanism for detecting the unexpected, in which informational value is defined by deviation from prediction (6, 7). Supernormal stimuli exploit this sensitivity by overwhelming novelty detection and symbolic salience systems. Examples include social media, which amplifies *Thumos* and *Agonix* through hyperactive status signaling and grievance tracking; pornography, which hijacks Venex by exaggerating novelty and decoupling sexual cues from pair bonding (42, 96–98); online radicalization, which overactivates *Sacrifex* by framing moral transcendence and martyrdom as heroic imperatives (9, 49); and extreme fitness or pro-ana cultures—and, in more valorized historical contexts, philosophical traditions such as Stoicism—fuse Sacrifex and Navigia by glorifying austerity, overcontrol, and symbolic purification through bodily discipline (98, 99).

These cultural vectors do not operate peripherally. They enter the behavior equation through C (Culture), altering the threshold field (Φ) and amplifying drive salience (D). Such distortions reveal that ARCH systems rarely fail due to structural deficits alone—they are dysregulated through interaction with symbolic culture. In this model, culture modifies Φ by altering symbolic salience. This modulation can produce behaviors that remain coherent in form but maladaptive in function, such as ritualized compulsions or culturally reinforced self-starvation.

Initially, this may result in ritualized but misaligned behavior—coherent in form but maladaptive in function. Over time, however, second-order biological consequences often emerge: nutrient depletion, endocrine disruption, neurotransmitter imbalances, and chronic stress. These changes feed back into the system, modulating Drive (D), reshaping threshold dynamics (Φ), and even altering access to archetypal scripts (A).

Consider another example in which symbolic misalignment initiates a cascade of biological dysregulation. Anorexia nervosa may begin with culturally amplified ideals of thinness (C), yet progressively lead to hypogonadism, serotonergic depletion, and altered amygdala reactivity—reshaping Drive (D) and Threshold (Φ) across systems. Chronic ideological activation, as seen in radicalization, may elevate *Thumos* and *Sacrifex* scripts, but also result in sleep disruption, cortisol elevation, and limbic sensitization, increasing vulnerability to threat- and grievance-based responses. In substance use disorders, culturally mediated expectations of euphoria, rebellion, or social belonging may initially activate *Venex* or *Thumos*. However, long-term exposure reshapes dopaminergic pathways, impairs executive function, and narrows motivational salience toward addictive cues—eventually distorting both D and Φ.

In such cases, what begins as a symbolic distortion becomes a systems-level disorder, where conserved neural architectures are not merely overactivated—they are retrained by cultural vectors and somatic feedback. The ARCH × Φ model is designed to capture this recursive architecture: Structure (A), Drive (D), Culture (C), and Threshold (Φ) do not operate in isolation or linear sequence—they form a dynamically interacting system. Culture can shape behavior, but behavior, once shaped, can shape the brain and ultimately harm the body and society in return (81). Having examined archetypes as structural scripts, drives as energetic amplifiers, and culture as symbolic modulators, we arrive at the final component of the equation: the threshold parameter (Φ). Thresholds determine not just what behavior is possible, but when and under what conditions it becomes expressed. They act as the gating function of the system, converting potential into execution. In the next section, we formalize Φ as a context-sensitive field, showing how cultural salience, neurobiology, and physiological states converge to regulate the probability of behavioral release.

## 10 Threshold (Φ): cultural modulation of activation

In the ARCH × Φ model, Φ represents a behavioral threshold field—the moment at which latent archetypal scripts, energized by internal drive (D) and modulated by cultural meaning (C), cross into overt behavior. Φ is not a simple arousal index; it is a context-sensitive gating function, shaped by both neurobiology and symbolic significance. The Φ parameter can be understood in thermodynamic terms as a gating function that reduces entropy within the behavioral system by reconciling multiple competing motivational signals.^7^ Crossing threshold Φ converts uncertainty into a coherent output. In adaptive states, Φ calibrates the balance between internal drives and external cues, yielding energy-efficient behavioral execution. In pathological states, dysregulation of Φ produces distinct entropy failures: entropy collapse (e.g., compulsive rituals, behavioral inhibition, catatonia) or loss of regulation (e.g., mania, impulsivity). In this sense, ARCH × Φ reframes psychiatric pathology as a failure of entropy regulation across motivational, cultural, and neurobiological domains.

Culture modulates Φ by altering the perceived salience, legitimacy, and urgency of a given behavioral script. As previously discussed, supernormal stimuli elicit exaggerated fixed responses (13, 24). Symbolic cues—such as ideological myths, purity codes, or moral absolutes—can lower Φ for specific archetypes, leading to premature, exaggerated, or dysregulated activation (9, 42, 43). Conversely, symbolic inhibition—through shame, ritual suppression, or narrative coding—can raise Φ, delaying or blocking expression even when the internal circuitry is prepared for activation.

Historical examples show that culture can regulate behavioral likelihood by modulating Φ directly. In totalitarian regimes such as Nazi Germany, collective propaganda lowered the threshold for Hitler to be seen as a *Hero* and *Savior* archetypes, while raising Φ for dissent and caregiving (9, 10). Similarly, in Milgram's obedience experiments, cultural authority cues reduced the Φ required for some participants to engage in behavior contrary to individual moral schemas (100). Thus, group effects recalibrate thresholds, making some behaviors easier and others more difficult to enact depending on context.

Trauma also alters Φ through neurodevelopmental recalibration. Early-life abuse can sensitize the Fear (*Phobon*) and Rage (*Agonix*) circuits via the HPA axis, while simultaneously raising the threshold for affiliative systems like *Theromata*. Over time, such modulation becomes embodied, affecting behavior even in the absence of overt cues, such as borderline personality disorder (81, 101–103).

In this model, culture is not merely expressive; it is causally active. It reshapes the neurocognitive environment in which behaviors are initiated. It redefines what feels urgent, permissible, or sacred, thereby altering the internal criteria for action. Whether through collective ideology or interpersonal trauma, symbolic context becomes a threshold-regulating field.

### 10.1 Dynamic modulation of Φ: neurobiological and clinical factors

While Φ has been described as a context-sensitive threshold governing behavioral activation, a more granular account of its modulation is warranted to support empirical operationalization. Φ is not a static parameter; it is a dynamic, biologically mediated construct influenced by internal physiological states, affective valence, and external environmental demands.

Neurobiologically, threshold sensitivity is modulated by multiple factors such as glucocorticoid tone (e.g., cortisol), monoaminergic signaling (e.g., serotonin, dopamine), and sleep-wake homeostasis, among others. For instance, elevated cortisol levels during acute stress can lower the Φ threshold for threat-detection archetypes (Phobon, Agonix), resulting in defensive reactivity even to ambiguous stimuli. Conversely, serotonergic depletion—as postulated in depressive states—may raise Φ globally, leading to behavioral inertia despite intact archetypal scripts and symbolic cues (88).

Fatigue, sleep deprivation, and chronic inflammatory states may also dysregulate Φ through disrupted hypothalamic-pituitary signaling and altered prefrontal-limbic integration. These physiological variables influence the salience and gating of archetypal expression, determining whether a behavioral script remains latent or is activated. Clinical examples include the blunted threshold for irritability or impulsivity in sleep-deprived individuals (lowered Φ for Agonix), or heightened thresholds for caregiving and affiliative behavior in postpartum depression (elevated Φ for Theromata) (81).

## 11 Adaptive learning: refinement of thresholds and scripts

Learning and habituation further refine the value of Φ over time. Repeated activation of specific archetypal systems can recalibrate threshold parameters through neuroplastic processes, such as the potentiation of hippocampal-prefrontal circuits in the context of trauma or dopaminergic reinforcement in performance-driven contexts (99–102). This recalibration may be adaptive or maladaptive, depending on the symbolic context and environmental feedback shaping Φ tuning. Importantly, these dynamics may also be quantifiable through established psychometric frameworks.

In this regard, personality models provide a valuable framework for understanding individual variability in threshold modulation. Such traits are both neurobiologically grounded and developmentally plastic, making them plausible markers of Φ sensitivity across archetypal domains. For example, elevated harm avoidance may increase Φ for threat-expressive archetypes such as Phobon, whereas high self-transcendence may reduce Φ for Sacrifex-related scripts, particularly under conditions of symbolic framing. These dynamics parallel Cloninger's distinction between temperament and character traits (103). Conceptualizing Φ as a modifiable, trait-linked threshold—rather than a fixed scalar—opens the possibility for personalized interventions that act on neurobiological, behavioral, and symbolic substrates to recalibrate adaptive behavioral readiness. Adaptive learning arises from the iterative modulation of archetypal thresholds through experience, feedback, and cultural encoding. This process is central to Dynamic Archetypal Coordination (DAC), in which multiple systems become entrained and optimized over time through recurrent activation and context calibration.

Coalition-building offers a further example of DAC: the integration of Thumos (recognition), Theromata (affiliation), and Sacrifex (shared purpose) creates durable social identities. These composites are governed by salience-weighted activation, stabilized through monoaminergic valuation systems and narrative framing (5, 50).

This recursive refinement process illustrates that thresholds are not fixed; they are conditioned by symbolic exposure and emotional experience, which determine not only what behaviors are enacted but what feels right, possible, or morally necessary (59–62). The result is not merely action but identity-embedded behavior, shaped by archetypal inheritance and cultural calibration.

### 11.1 Metaplasticity and ARCH modulation

While neuroplasticity refers to changes in synaptic strength and circuit activation in response to experience, metaplasticity describes changes in a system's potential to change—that is, the modulation of plasticity thresholds themselves. This concept is critical to understanding how the ARCH × Φ model incorporates long-term adaptation and symbolic reframing. Repeated activation of archetypal systems can lead to synaptic strengthening through mechanisms such as long-term potentiation (LTP), particularly within the hippocampus and cortical association areas (82). This plasticity not only reinforces behavioral scripts but may lower the Φ threshold required for their future activation, making certain roles or responses more easily accessible. Over time, symbolic rehearsal and emotional salience further shape these circuits, modulating their reactivity and potential for reactivation. Metaplasticity (104, 105) allows archetypal systems to become more or less recruitable over time. For example, repeated caregiving under stress may lower the activation threshold (Φ) of the Theromata system, making caregiving scripts more reflexively accessible. Conversely, repeated threat exposure may heighten the Φ threshold of Phobon, leading to delayed or blunted defensive responses.

Molecularly, metaplasticity involves mechanisms such as NMDA receptor subunit switching, changes in neuromodulatory tone (e.g., serotonin, dopamine), and homeostatic scaling in cortical circuits. Neurosteroids, such as allopregnanolone and DHEA, influence excitability by modulating the GABA-A and NMDA receptors, thereby affecting learning, fear extinction and emotional regulation (105, 106).

These adjustments reflect not just learned behavior but learned learnability—how quickly a system can be reactivated in the future. In symbolic systems, metaplasticity interacts with cultural framing and identity scripts; roles that are practiced, ritualized, or socially reinforced become more easily accessible over time, even if they were initially effortful. This allows for the entrenchment of professional roles, ideological stances, or moral behaviors as stabilized archetypal patterns.

## 12 Probabilistic templates and neurobiological mediation

In the ARCH × Φ model, archetypes are not deterministic programs. They are probabilistic neural templates, organized around modular circuits that can be activated, inhibited, or recombined depending on internal and external conditions (3).

These scripts unfold in phased modulation rather than binary logic:

1. Latent readiness based on phylogenetic architecture (A),
2. Drive-dependent energizing (D),
3. Cultural-symbolic modulation (C),
4. Threshold crossing (Φ), dependent on cumulative salience.

Neuroanatomically, these stages are mediated by subcortical circuits (e.g., hypothalamus, amygdala, periaqueductal gray) for initiation, and by cortical networks (e.g., prefrontal cortex, default mode network) for symbolic integration and inhibition (15, 82). These systems interact with hormonal and monoaminergic inputs (e.g., dopamine, serotonin, oxytocin), which influence the attribution of salience and the arbitration of archetypes (48, 50, 76, 88).

Scripts may co-activate, compete, or shift over time. For instance, in a morally ambiguous context, Agonix (competitiveness) may be tempered by Theromata (care) or Sacrifex (purpose), depending on symbolic framing and emotional resonance. These interactions are resolved probabilistically through dopaminergic modulation and narrative constraints, some of which have been analogized to radio dials (5, 42).

This logic extends to cross-species cultural transmission. Ethological studies have demonstrated that primates and other mammals exhibit ritual-like behaviors, tool use, and social customs, indicating that culture influences the expression of archetypal traits across species (27, 67). Examples include chimpanzee termite fishing, regional vocal dialects in birds, and cooperative hunting in orcas—each a symbolic elaboration of conserved behavioral archetypes.

Humans amplify this capacity through the use of symbolic abstraction. We encode archetypes into institutions—such as education, law, and religion—and tether behavior to culturally defined meanings. Trauma may encode fear-based scripts; sacred ritual may elevate sacrifice into identity. These mappings are mediated at least in part by the Default Mode Network (DMN), which is thought to support autobiographical coherence, narrative integration, and symbolic moral reasoning (107, 108).

Over time, cultural symbols shape which behaviors are perceived as virtuous, threatening, or redemptive. The brain does not change, but its output does, because the symbolic field has shifted.

## 13 Culture as modulation in ARCH × Φ

Culture is not a surface adornment over biology—it is an interpretive partner and behavioral amplifier. In the ARCH × Φ model, behavior arises from the coordinated interaction of Archetype, Drive, and Culture, each contributing a distinct but converging causal role. Neural scripts (Archetypes) provide structure; motivational systems (Drive) supply energetic activation; and symbolic systems (Culture) shape meaning, salience, and behavioral trajectory. The ARCH equation thus provides a unified framework for understanding behavior as evolutionarily conserved, energetically driven, symbolically shaped, and threshold-regulated.

This logic is empirically tractable: in rodents, stress exposure alters the expression of the lordosis reflex—quantified by the Lordosis Quotient (frequency) and Intensity Scores (magnitude)—by recalibrating sexual receptivity circuits (A and D) and increasing thresholds (Φ) (20). In humans, early sexual trauma likewise reshapes threshold dynamics (Φ) and symbolic coding (C), producing long-term alterations in relational and sexual behavior (57). These canonical studies underscore that Culture, encompassing cues and contexts, is not an external overlay but a causal partner in sculpting behavioral trajectories.

A parallel can be drawn: just as fragments of arcs are perceptually "closed" into a circle under Gestalt rules, fragments of social cues can be integrated into an archetypal script when Drive (D), Archetype (A), and Culture (C) converge. For example, brief signs of vulnerability (facial expression, posture, tone of voice) may be insufficient in isolation, but when amplified by parental drive and cultural coding of caregiving, they "close" into the full Theromata caregiving script, lowering Φ and releasing nurturant behavior. When cues are misaligned or Drive is absent, the script remains latent, just as fragments of arcs without alignment fail to produce closure. As in a perceptual closure study (5), a behavioral "script closure" could be quantified by comparing the similarity of neural or behavioral responses to complete, aligned, and disordered cue sets, providing an empirical test of how Archetype, Drive, Culture, and Threshold converge.

## 14 Illustrations of the ARCH model

Postpartum caregiving: A parent responds to infant cries despite severe sleep deprivation. This is not simply moral discipline—it is the activation of the Theromata system, supported by oxytocin, sensory cues, and the Care drive. Cultural overlays (e.g., maternal identity, virtue norms) further amplify behavioral salience.

Academic overdrive: A high school senior develops a compulsive habit of checking grades. The Agonix vector is activated via competitive triggers, supported by Seeking and Fear drives. Culture elevates these signals, transforming routine evaluation into symbolic threat and identity pressure.

Generative mentorship: A retired professional volunteers as a youth mentor. This reflects the Sacrifex archetype, expressed through legacy and civic duty, reinforced by activation of the Theromata archetype. Cultural frames assign moral worth to self-transcendence and community building.

Courtship: At its foundation, reproduction begins with Navigia: sperm cells exhibit directional movement toward the egg, driven by ATP-fueled motility and guided by chemotactic gradients. Once embodied, courtship engages the Venex system, initiating sexual signaling, grooming, and display. But courtship is rarely purely reproductive. It is often playful (Hedonix), shaped by imitation (Imitati) of culturally scripted norms, and modulated by Thumos, as individuals compete for recognition, prestige, or favor. In longer-term relationships, courtship is sustained by Theromata (affiliative bonding) and often invokes Sacrifex—ritual gestures, gifts, or symbolic vulnerability. In collective contexts (e.g., weddings), Alligantia may be activated, as alliances are socially sanctioned.

Each of these patterns reflects the coordinated activation of multiple archetypal systems, modulated by internal drive states and filtered through symbolic meaning. From caregiving to courtship, the expression of behavior arises not from a single neural script, but from the dynamic interplay of evolutionarily conserved architectures shaped by context, identity, and culture. While the preceding illustrations highlight adaptive and normative expressions of ARCH dynamics, the same framework can also illuminate clinical and forensic contexts. When archetypal scripts are excessively amplified, distorted by cultural salience, or dysregulated by drive and threshold failures, behavior may shift from adaptive coherence to maladaptive compulsion or pathology. In the following section, we apply the ARCH × Φ grammar to clinical case material, demonstrating how psychiatric syndromes and symbolic fixations can be reframed as dysregulated archetypal activation rather than categorical symptom clusters.

## 15 Clinical case examples: maladaptive archetypal activation

In this section, we illustrate how the ARCH × Φ model can help interpret psychopathology not as the breakdown of behavior, but as the maladaptive amplification or distortion of otherwise adaptive archetypal systems. Each case reflects dysregulation across A (archetypal pattern), D (drive amplification), and C (cultural framing), resulting in dysfunctional or compulsive scripts. The cases are presented here as illustrative applications of the ARCH × Φ grammar and intended to demonstrate how the computational framework might be used to interpret psychopathology.

Case example: Restrictive Eating and Symbolic Control.

An adolescent develops progressively restrictive eating behaviors framed around ideals of thinness and purity. While superficially a matter of dietary restraint, this case illustrates how ARCH × Φ organizes psychopathology as dysregulated archetypal activation.

• Archetype (A):
 o Agonix (competition/status striving) — competitive self-comparison and perfectionistic monitoring.
 o Sacrifex (self-transcendence/altruism) — valorization of bodily sacrifice framed as discipline or purity.
 o Navigia (goal-seeking/exploration) — channeling curiosity and mastery into calorie tracking, exercise regimens, and body measurement.
• Drive (D):
 o Fear drives (threat of weight gain, social rejection) interact with seeking drives (reward from self-control).
 o Dopaminergic and serotonergic modulation sustain compulsive focus, with intermittent hedonic reinforcement when goals (weight milestones) are achieved.
• Culture (C):
 o Thin-ideal media and "pro-ana" online communities supply supernormal stimuli that exaggerate culturally valued cues of slenderness.
 o These symbolic exaggerations function as metadata tags, amplifying the salience of thinness cues and framing them as moral imperatives (purity, virtue, control).
• Threshold (Φ):
 o Repeated cultural amplification lowers Φ for Sacrifex- and Agonix-driven behaviors, making restrictive rituals more readily deployed.
 o Over time, malnutrition further reduces serotonergic tone, reinforcing low Φ thresholds and narrowing behavioral repertoires (Fixed Archetypal Action Patterns).

Behavioral output:

The adolescent's restrictive eating emerges not from "choice" or a single causal factor, but from the convergent activation of multiple archetypes (A), energized by fear- and reward-related drives (D), distorted by cultural metadata tags (C), and gated through a lowered threshold (Φ). The behavior persists as a compulsive archetypal script—coherent in form, but maladaptive in function. This process parallels Lorenz's imprinting in birds, where attachment scripts become fixed when drives and cues converge during critical windows (36, 77). In adolescence, when identity scripts are highly plastic, exposure to thin-ideal cultural cues can act as symbolic imprinting: conserved archetypal motifs of competition (Agonix) and sacrifice (Sacrifex) become rigidly tagged to body image, lowering Φ for restrictive behaviors. Once established, this imprinting shapes long-term motivational patterns, even in the absence of continued exposure. Indeed, weight loss and dieting behavior have been described as "coded" rewarding, with the amygdala, ventral striatum, and orbitofrontal cortex all engaged (109).

## 16 Functional dynamics

**Figure 1:**
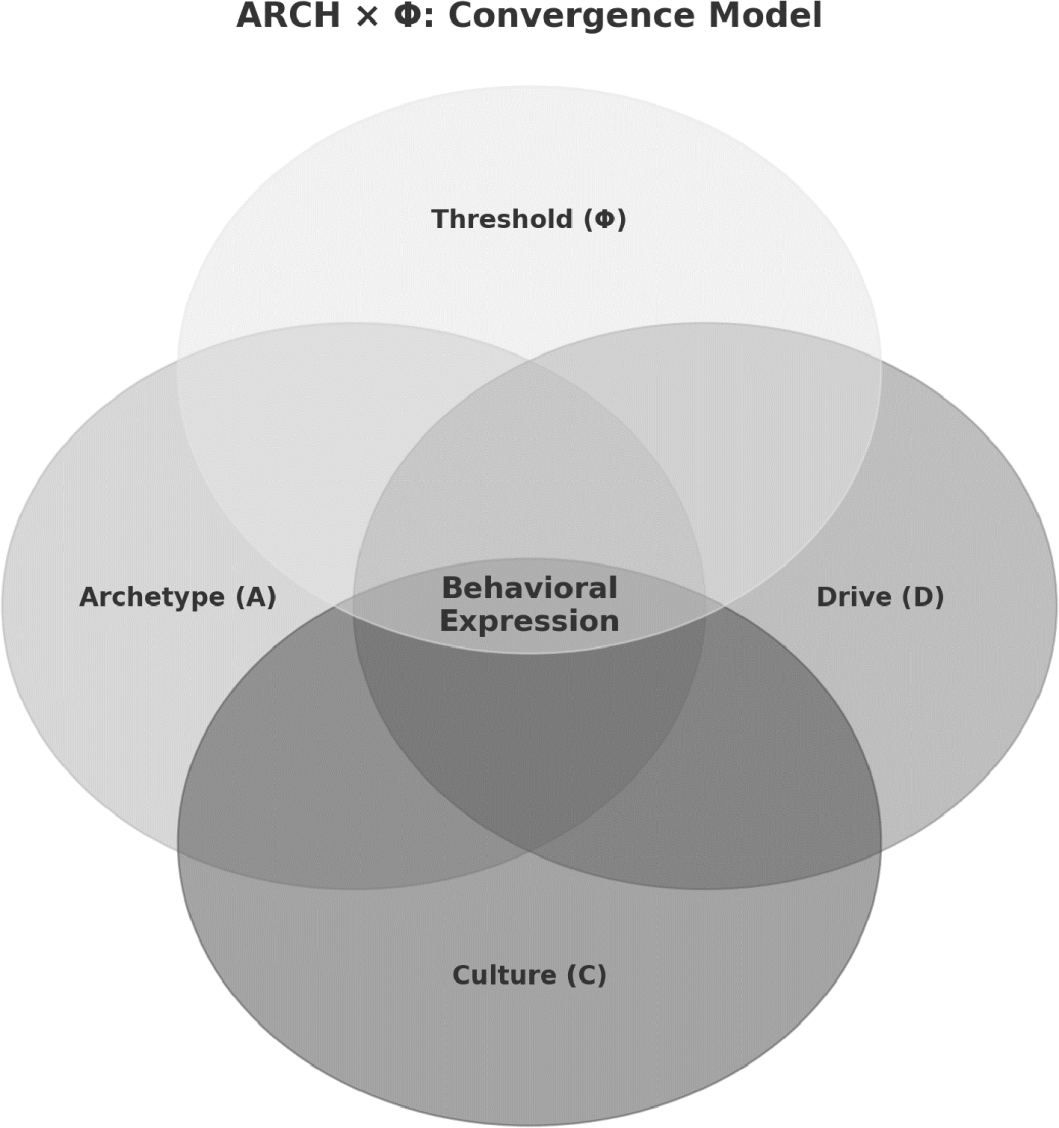
ARCH × Φ convergence model. A four-circle Venn diagram illustrating the conjunctive logic of the ARCH × Φ framework. Archetype **(A)** provides the conserved neural script, Drive **(D)** supplies motivational energy, Culture **(C)** assigns symbolic salience, and Threshold **(Φ)** gates context-sensitive release. Behavioral expression occurs only when all four domains overlap; the absence of any one component suppresses output.

The ARCH structure can be visualized in a Venn diagram, illustrated in **Figure 1**.

The ARCH model was initially illustrated as a classical stone arch, with Archetype (A) and Drive (D) forming the columns, Culture (C) serving as the keystone, and Threshold (Φ) as the base. This metaphor emphasizes structural interdependence; however, we present a Venn diagram to more directly illustrate the convergence logic of ARCH × Φ. This structure emphasizes that behavior is not reducible to any single factor, but emerges from the convergence of neural potential, energetic salience, and cultural meaning.

When these components fall out of balance, the model allows for quantifiable descriptions of pathology:

- Drive/Archetype > 1: unmodulated behavior (e.g., compulsivity, aggression, addiction).
- Culture/(A × D) ≫ 1: symbolic rigidity, overvalued beliefs (45), hyperconformity.

ARCH's ten provisional core systems—*Navigia, Theromata, Phobon, Agonix, Sacrifex, Thumos, Imatati, Alligantia, Venex*, and *Hedonix*—form the neural basis for behavior across diverse contexts. Their interaction is governed by Dynamic Archetypal Coordination (DAC), a process by which these systems activate, inhibit, or entrain one another in real-time. The result is a unified model of behavior: structured, energized, symbolic, and recursively shaped by feedback. DAC explains not only what people do, but why they do it, when, and under what conditions behavior changes.

## 17 Diagnostic paradigms

Despite numerous iterations, the Diagnostic and Statistical Manual of Mental Disorders (DSM) remains a symptom-based classification system. It categorizes behavioral presentations but offers little explanatory insight into how or why these behaviors emerge (81). Specifically, it omits the neural architectures and symbolic meaning structures that shape human motivation and behavior. In contrast, the ARCH model defines behavior as a triadic product of conserved neural scripts (Archetypes), dynamic biological energy (Drive), and culturally embedded meaning (Culture). This framework enables both bottom-up (neuro-affective) and top-down (cultural-symbolic) pathways to be modeled in parallel (110).

The Research Domain Criteria (RDoC) framework similarly seeks to ground psychiatric diagnosis in neurobiological mechanisms. However, RDoC's behavioral domains are often detached from phylogenetic context and fail to account for symbolic culture (111). ARCH extends RDoC by restoring evolutionary continuity and cultural encoding, providing a richer interpretive map of how conserved behavior systems become distorted or adaptive in modern contexts.

Instead of describing surface symptoms, ARCH supports structural-behavioral phenotyping: Which archetypes are activated? What drive states are dominant? How is cultural encoding shaping, amplifying, or distorting behavior? How do medications, group effects, and psychotherapy align within this causal framework? These questions may allow clinicians to move beyond categorical diagnoses toward understanding structure, motivation, and meaning in the context of individual patients.

## 18 Validation pathways

To gain scientific traction, ARCH must be empirically testable. Canonical behavioral motifs—such as caregiving, threat defense, status competition, and sacrificial behavior—are already supported by comparative ethology. Neurobiological studies identify conserved substrates: the medial preoptic area (mPOA) in caregiving (15, 46), periaqueductal gray (PAG) and hypothalamus in defense (42, 48), and mesolimbic dopamine circuits in competition and reward (51, 52). The DAC framework—real-time coordination of multiple archetypes—resembles Buzsáki's oscillatory logic (79), where neural motifs phase-lock to generate coherent output. DAC may represent a behavioral-level analogue of such neurodynamic coordination. ARCH therefore generates clinically testable questions: Are certain behaviors due to overactive archetypes (A), distorted drives (D), cultural amplification (C), or their combinations? How do symbolic scripts modify Φ and shift behavior over time? Can narrative reframing, hormonal modulation, or exposure reshape DAC? Initial case studies (e.g., martyrdom, OCD rituals) suggest theoretical coherence and translational relevance. Formal validation will benefit from integration of neuroimaging, ethological modeling, and narrative coding across cultures and the lifespan.

## 19 Experimental paradigms for empirical testing

The falsifiability of ARCH × Φ can be pursued through experimental paradigms that selectively manipulate archetypes, drives, and cultural priming. For example:

- Fear conditioning paradigms to probe Phobon thresholds under varying cultural frames (e.g., symbolic threat narratives vs neutral stimuli).
- Parental caregiving tasks (e.g., infant cry paradigms) to test Theromata activation under oxytocin modulation.
- Competitive games (e.g., iterated prisoner's dilemma, dominance challenges) to measure Agonix activation, with fMRI mapping of VTA–NAc projections.
- Cultural priming studies (e.g., exposure to honor-related vs neutral cues) to examine modulation of Thumos thresholds.

Comparable approaches have already been implemented in computational psychiatry. For example, Montague and colleagues (112) have used economic game paradigms (e.g., trust and ultimatum games) in conjunction with hyperlinked neuroimaging to model aberrant valuation and social decision-making in psychiatric illnesses such as borderline personality disorder. This paradigm illustrates how computational models of human motivation can bridge behavioral economics, neural circuits, and psychopathology, providing a translational precedent for ARCH × Φ validation. Such paradigms allow for clear predictions: behavior should emerge only when archetypal structure, drive intensity, and cultural cues converge sufficiently to cross Φ; absence of any factor predicts behavioral suppression.

Recent evidence further supports this line of reasoning from the perspective of Bayesian priors. A brain-wide imaging study in mice (113) demonstrated that prior information is distributed across sensory, motor, and associative regions (Archetype, A), updated dynamically from recent actions via an exponential kernel (Drive, D), and gated by the hidden probability structure of the task (Culture, C).^8^ Expression occurred as a baseline readiness signal modulated by global brain state (Φ). These findings align directly with ARCH × Φ, illustrating that probabilistic inference in the brain requires conjunctive convergence across conserved circuitry, motivational drive, contextual coding, and threshold regulation. A modification of this paradigm could directly test ARCH × Φ: by selectively perturbing one component—such as reducing Drive via dopaminergic blockade, altering contextual coding through reframed probability structures, or modulating Φ with arousal-state manipulations—researchers could determine whether priors fail to emerge when any single factor is absent, thereby falsifying the model.

Taken together, these paradigms and findings establish a foundation for deriving specific, falsifiable hypotheses, which we outline in the next section (19.2). Yet several empirical questions remain open. A key step will be to establish the inter-rater reliability of archetypal coding, much as diagnostic systems report agreement metrics. No systematic studies have yet quantified whether independent raters converge in identifying the same archetypal patterns in behavior or narrative. Another question concerns the distribution of archetypes within individuals: while all ten systems are available as latent neural schemas, most people likely express a limited subset of dominant archetypes, shaped by temperament, hormonal tone, development, and cultural reinforcement. Finally, the stability of expressed archetypes over time requires longitudinal study. Archetypal schemas are conserved as latent motifs, but their expression shifts with changing drives, thresholds, and cultural contexts. Some archetypes crystallize into enduring identities (e.g., Healer, Warrior), while others remain situational. Addressing these issues—reliability, dominance, and stability—will be crucial for empirical validation and clinical utility.

### 19.1 Retrospective vs. prospective application

At present, the ARCH × Φ framework is most powerful when applied retrospectively. It can map observed behaviors—such as martyrdom, targeted violence, compulsive caregiving, or victimhood—back onto the conserved archetypal systems from which they arise. This retrospective application is clinically and forensically useful, as it clarifies which archetypal substrates were engaged, which drives were amplified, and which cultural cues lowered threshold Φ, thereby reconstructing the motivational structure of behavior after the fact. The model is also designed for prospective testing, but this remains a goal rather than an established capacity. In principle, systematic measurement of archetypal sensitivity (e.g., neuroimaging, lesion studies), drive states (e.g., hormonal or neuromodulatory assays), cultural salience (e.g., narrative priming), and threshold dynamics (e.g., stress or arousal modulation) should allow estimation of the likelihood of particular archetypal scripts (e.g., Warrior, Martyr, Healer) emerging under specified conditions. In this way, ARCH × Φ seeks not only to explain past behaviors but also to evolve into a predictive grammar of human action that can be subjected to empirical falsification.

Falsifiability of ARCH × Φ.

To highlight falsifiability, each archetype generates both confirmatory and disconfirmatory predictions. For example, in female rats, lesions of the VMHvl or mPOA abolish lordosis in hormonally primed rodents despite intact drive and cues—a confirmatory prediction consistent with the multiplicative logic (1 × 1 × 0 = 0). If lordosis were to persist after such lesions, the model would be disconfirmed. Similarly, mPOA lesions abolish maternal caregiving (Theromata); amygdala lesions abolish conditioned fear (Phobon); and ACC lesions reduce honor-restoring behaviors (Thumos). Each case demonstrates that ARCH × Φ is not descriptive alone, but testable: if behavior survives in the absence of its archetypal substrate, the model requires revision.

Using Venex as an example, the following confirmatory or falsifiable outcomes are predicted by ARCH × Φ:

- A → 0 (VMHvl or mPOA lesion): lordosis absent despite estrogen and male cues.
- D → 0 (no estrogen priming): lordosis absent despite intact circuit and cues.
- C → 0 (no male/cutaneous cues): lordosis absent despite circuit and estrogen.
- ↓Φ (e.g., amphetamine intoxication): collapses threshold gating, leading to maladaptive overexpression of Venex archetypal vector script.

### 19.2 Proposed empirical predictions and hypotheses

Operationally, the ARCH × Φ equation can be expressed as a probabilistic function, such that behavior emerges when the joint influence of archetypal vectors (A), Drive (D), and Culture (C) surpasses the threshold (Φ). In practice, this can be modeled using logistic or softmax functions, allowing for the estimation of salience weights and threshold parameters in experimental paradigms. To facilitate empirical validation, we outline a set of falsifiable hypotheses derived from the ARCH × Φ framework. These predictions span neurobiological, behavioral, and symbolic domains, and are amenable to experimental, neuroimaging, and clinical methodologies.

1. Archetypal Impairment Suppresses Behavior Despite Intact Drive and Culture.
 Prediction: Lesions or functional disruptions in archetype-linked circuits (e.g., medial preoptic area (mPOA), periaqueductal gray (PAG), anterior cingulate cortex (ACC)) will suppress the associated behavioral output even when drive and cultural cues are present.
 Test: Use of lesion models or targeted neuromodulation (e.g., transcranial magnetic stimulation (TMS), optogenetics) in animal or human subjects to observe suppression of caregiving, threat response, or sacrificial behavior under high drive conditions. Genetic and epigenetic manipulations provide parallel strategies; for example, estrogen receptor knockouts abolish lordosis behavior (Venex), while variants of oxytocin or vasopressin receptors alter affiliative and bonding behaviors (Theromata). These approaches allow A (archetypal substrates) and D (drive systems) to be mapped to specific genomic domains, offering a molecular test of the model.
2. Modulation of Φ Alters Behavioral Activation Thresholds.
 Prediction: Pharmacological or contextual modulation of Φ (e.g., via cortisol elevation or symbolic priming) will shift the threshold for archetypal behavior, independent of changes in A, D, or C.^9^.
 Test: Manipulate stress or symbolic salience in controlled environments (e.g., using social threat cues or moral narratives) while measuring behavioral response latency or neurophysiological readiness.
3. DAC Instability Predicts Maladaptive Composite Behavior.
 Prediction: High drive states (D) combined with conflicting archetypal activations (e.g., Agonix + Sacrifex) will result in maladaptive or rigid behavior patterns, particularly when Φ thresholds are lowered.
 Test: Simulate high-stakes decision tasks under affective load (e.g., moral dilemmas) and assess archetypal coactivation using fMRI or network connectivity analysis.
4. Symbolic Reframing Alters Culture (C) and Modulates Behavior.
 Prediction: Narrative interventions that reframe symbolic meaning will shift C and either amplify or inhibit archetypal scripts, even in the absence of neurobiological change.
 Test: Employ narrative-based therapy or symbolic revaluation paradigms and measure shifts in archetypal salience or activation (e.g., reduced Phobon expression following reframing of threat).
5. Archetypal Salience Predicts Role-Constrained Behavior Across Cultures.
 Prediction: Canonical archetypal systems (e.g., Theromata, Thumos) will exhibit conserved neurobiological activation patterns across cultures, even when their symbolic and behavioral expressions diverge. For example, caregiving roles may appear as maternal caregiving in one society and elder devotion in another, yet recruit homologous neural substrates (e.g., anterior insula, hypothalamus, oxytocinergic pathways). Similarly, honor-based behavior may vary in surface norms but consistently engages regions implicated in moral salience, recognition, and status processing.
 Test: Cross-cultural fMRI paradigms assessing archetypal behaviors—such as caregiving, sacrifice, or honor-defense—should reveal common activation in conserved circuits, despite cultural variation in role framing or symbolic input. Experimental designs may require within-subject controls, long scan times, or advanced modeling (e.g., representational similarity analysis) to capture individualized threshold shifts and multi-system activation.

Sample Considerations: Group-level comparisons will likely require a large, demographically diverse sample size to overcome symbolic variance and permit generalization. Precision interventions may benefit from idiographic modeling frameworks, wherein individuals function as their baseline across systematically varied, culturally framed contexts, allowing for fine-grained inference about threshold modulation and symbolic sensitivity.

These hypotheses are explicitly falsifiable: if lesions fail to suppress archetypal behavior, if threshold manipulations do not shift activation probability, or if symbolic reframing leaves behavior unchanged, then the ARCH × Φ framework would require revision.

### 19.3 Operational definitions

To increase testability, several abstract constructs within the ARCH × ϕ framework can be operationalized with convergent measures (1). Identity fusion—the perceived equivalence of personal and group identity—can be indexed with self-report scales (e.g., pictorial and verbal fusion measures), costly pro-group choice tasks, and fMRI responses in mPFC–TPJ networks during in- vs out-group dilemmas (2). Grievance salience, defined as the weighting of humiliation or injustice cues, may be measured through inequity paradigms (e.g., ultimatum or exclusion tasks), state anger/hostility scales, insula–ACC reactivity, and hormonal markers such as testosterone and cortisol ratios (3). Symbolic distortion, or the rigid attribution of meaning to purity, threat, or ideological cues, can be assessed with belief-updating tasks (e.g., BADE)^10^, narrative analysis of speech samples, moral purity scales, and fMRI recruitment of DMN and amygdala during symbolic primes. This approach is supported by recent evidence that politically extreme individuals exhibit similar neural processing despite ideological differences (111). This convergence supports the attractor logic: different surface narratives can still engage conserved neural motifs (28, 29). In ARCH × ϕ terms, the archetypal attractor is conserved, while cultural content provides variable surface expression In ARCH terms, these measures provide candidate indices of salience weights, drive amplification (D), cultural tagging (C), and threshold modulation (Φ), allowing translation of theoretical constructs into empirically testable variables.

## 20 Neurodynamics of internal and external behavior

ARCH aligns with emerging neuroscience, showing that the brain oscillates between internal narrative simulation and external goal pursuit. The Default Mode Network (DMN)—involving the medial prefrontal cortex, PCC, and TPJ—is active during reflection, symbolic processing, and archetypal rehearsal. It supports self-narrative, moral modeling, and role imagination—key elements of cultural scripting and archetype modulation.

Conversely, the Action Mode Network (dorsolateral PFC, dorsal ACC, cingulo-opercular regions) governs attention, planning, and task execution. ARCH proposes that behavioral enactment reflects dynamic switching: a movement from internal archetypal modeling (via DMN) to external performance (via action networks). This provides a neurodynamic mechanism for DAC: the brain does not just "decide"—it navigates between symbolic potentials and enacted outputs (107, 108).

## 21 Model refinement and theoretical expansion

ARCH remains a generative theory—conceptually fertile, but in need of operationalization. Archetype scripts (A) may be indexed via functional magnetic resonance imaging (fMRI), positron emission tomography (PET), or behavioral proxies (e.g., medial preoptic area (mPOA) activity during empathy tasks; periaqueductal gray (PAG) during fear; ventral tegmental area (VTA) during status challenges). Drive (D) can be measured through converging proxy indicators, such as neuroendocrine markers (e.g., cortisol, testosterone), psychophysiological states, behavioral effort tasks, and validated self-report instruments such as the Positive and Negative Affect Schedule (PANAS) or Temperament and Character Inventory (TCI) (103). Culture (C) can be quantified using Hofstede's scales, Schwartz's value frameworks, and narrative analysis. Threshold (Φ) might be inferred through response latency, salience ratings, or physiological arousal during symbolic tasks. Meaning-making itself can be conceptualized as a thermodynamic regulator: symbolic coherence reduces informational entropy by aligning behavior with culturally sanctioned roles and narratives. In this sense, culture does not merely "overlay" behavior but directly participates in entropy regulation, shaping both the likelihood and the energetic cost of behavioral expression. ARCH can be integrated into RDoC as a multi-level explanatory framework, connecting affective circuitry, behavioral motifs, and symbolic meaning systems. Over time, the model could be expanded to include oscillatory coupling (e.g., Buzsáki), narrative timescales (e.g., Varela), or temporally extended symbolic cognition (e.g., Bergson, Dennett). These expansions may support computational modeling and cross-disciplinary collaboration. Beyond clinical psychiatry, the explanatory reach of ARCH × Φ extends into forensic and security domains. The same archetypal grammars that organize caregiving, competition, or sacrifice can, under conditions of drive amplification and symbolic distortion, be co-opted into violent trajectories. In this sense, targeted violence and terrorism may be understood not as anomalies, but as maladaptive expressions of conserved scripts. We therefore turn next to the forensic application of the model, illustrating how ARCH × Φ can decode ideologically motivated aggression and inform threat assessment.

## 22 Forensic application: archetype killers

There is a pressing societal need to understand better and prevent ideologically motivated violence, which remains a persistent threat in schools, workplaces, communities, and geopolitical contexts. Current approaches to threat assessment are often descriptive, focusing on surface behaviors or risk factors, but lack a unifying grammar to explain *why* specific sure, but the audience is very high level com individuals escalate to violence while others do not. The ARCH × Φ framework offers a translational lens for this problem, grounding violent behavior in conserved neural systems, motivational drivers, and symbolic framing. Composite Archetypes—such as Avenger + Martyr + Defender—often appear in offenders with extreme overvalued beliefs (EOBs) (9, 62). These individuals are not psychotic; their behavior reflects overactivation of conserved neural scripts, amplified by symbolic grievance narratives.

In a recent study of 15 cases of targeted violence—including assassins, terrorists, and mass shooters—the ARCH framework was used to map dominant archetypal roles, motivational drivers (e.g., thymotic urgency), and symbolic rationales (e.g., grievance narratives, identity fusion) (9). This model, we termed *archetype killers*, helped distinguish violence rooted in psychotic delusion from that arising from narratively organized, ideologically fused belief systems. In several cases involving school shooters, the framework supported recognition of threshold dynamics, where internal activation states (rage, humiliation, symbolic grievance) crossed into operational planning and attack behavior (62, 63). These findings suggest that it is not the content of ideology that primarily drives radical behavior, but the emotional circuitry engaged during grievance, identity fusion, and symbolic framing (9, 114). In ARCH × Φ terms, this highlights the role of emotion in lowering threshold (Φ) across archetypal systems, making certain scripts more likely to activate regardless of political orientation. The common denominator may therefore be heightened affective salience, which drives script release across divergent ideological narratives. Forensic research may find ARCH helpful as a complement to such studies, clarifying symbolic identity, motivational structure, and cultural framing, thereby enhancing assessments of culpability, risk, and treatment potential (9, 62, 63).

To date, the application of ARCH to targeted violence and EOBs is limited to a single retrospective analysis in which we mapped behaviors onto underlying archetypal activations (9). The framework is, however, amenable to prospective testing, with the understanding that any forward-looking assessment will produce false positives and false negatives and therefore requires careful calibration, ethical safeguards, and empirical monitoring. A prospective design could enroll *high-risk cohorts* identified through standard threat-assessment criteria (e.g., school, workplace, or community threat assessment teams). Archetypal coding (A), drive indicators (D; endocrine and physiological proxies), cultural salience measures (C; grievance or identity fusion), and threshold proxies (Φ; arousal or response latency) could then be collected at baseline and longitudinally. Pre-registered predictions might test near-term outcomes such as escalation versus de-escalation. Model performance should be reported in terms that show both accuracy and practical usefulness. This includes measures of how well the model distinguishes actual cases from non-cases (e.g., sensitivity/specificity), how often predictions are correct (positive and negative predictive value), and whether independent raters agree on archetypal coding. Validation should also be done on new, independent samples to ensure generalizability. Because such work involves low base rates and carries a risk of stigmatization, it must be conducted under independent oversight, with minimal and proportionate data collection, robust privacy protections, and clear safeguards not to harm participants.

### 22.1 Thymotic drive: spiritedness and symbolic salience

As introduced in Section 3.1, thymos (or thumos) is derived from the Greek for spiritedness, pride, or moral self-assertion. Thymotic drive represents a valence-laden, cross-system amplifier that modulates archetypal activation in response to symbolic injury, perceived injustice, or threats to identity. It frequently augments the expression of Agonix (competition), Imitati (mimetic alignment), or Sacrifex (symbolic devotion) archetypes, depending on the context and narrative framing.

Thymotic drive is observed across species—particularly in primates—and has played a pivotal role in shaping human history. It underlies behaviors marked by recognition-seeking, moral protest, legacy-building, and sacrificial intensity. In contemporary contexts, it contributes to phenomena such as ideological violence, whistleblowing, copycat behavior, and symbolic protest, where motivation is less hedonic and more aligned with ethical or symbolic imperatives.

This construct is particularly salient in the formation and enactment of extreme overvalued beliefs (EOBs) and in the valorization of martyrdom and sacrificial warfare (9, 59). **Table 2** illustrates how thymotic drive may contribute to the coordinated activation of archetypal systems during intergroup conflict.

## 23 Limitations

ARCH × Φ, though integrative, remains theoretical and only partially validated. Its constructs—especially Φ and DAC—require further operationalization. Empirical studies are needed to test inter-rater reliability of archetypal coding, drive–archetype ratios, symbolic thresholds, and cross-cultural script modulation. While the model draws from Western science and mythic frameworks, future work must also explore non-Western symbolic systems and archetypal grammars. The formal behavior equation proposed by the ARCH × Φ model is conceptually generative, yet it has not been fully operationalized within computational or neurodynamic models. Future research should pursue neurodynamic simulations, longitudinal ethnographic analyses, and symbolic modeling to test and refine the model's parameters empirically. These efforts are crucial for developing ARCH as a translational tool that bridges neuroscience, psychiatry, psychology, and behavioral science. With further refinement, the framework may contribute to precision psychiatry, offering individualized insights into behavioral activation patterns and symbolic modulation. These limitations notwithstanding, the ARCH × Φ model is presented as a generative framework rather than a finished product. Its value lies in offering a structured grammar that can be tested, falsified, and refined across biological, cultural, and computational domains. In this spirit, we now reframe ARCH not only as a clinical and forensic tool, but also as a computational lens on the foundations of behavior itself.

## 24 Computational reframing of the foundations of behavior

Psychiatry often describes symptoms without causal explanation. In ARCH × Φ, behavior is modeled as the product of structure (A), energy (D), and meaning (C), released when conditions cross a threshold (Φ). Equally, ARCH × Φ affirms the mind–body connection: interoceptive signals—hormonal states, immune tone, autonomic rhythms—tune thresholds (Φ), linking physiology to symbolic meaning and archetypal expression (38, 42, 103).

## Data Availability

The original contributions presented in the study are included in the article/supplementary material. Further inquiries can be directed to the corresponding author.

## Footnotes

1 Tinbergen’s classic ethological experiments showed that male sticklebacks attack crude models with red undersides, illustrating how a simple visual cue can act as a releaser for a fixed action pattern (FAP). Lorenz similarly described how greylag geese will roll oversized artificial eggs into their nests, a “supernormal stimulus” that exaggerates the natural releaser.^13^ Building on such foundational ethological insights—and on subsequent work linking these mechanisms to symbolic psychology—we extend these concepts into the human domain. In our formulation, symbolic releasers are culturally encoded cues (e.g., myths, conspiracy theories, images, slogans) that trigger the activation of archetypal patterns. In contrast, symbolic imprinting refers to emotionally charged exposures during sensitive developmental windows that fix symbolic meaning into archetypal scripts. This extension reflects our deliberate application of ethological principles to human cognition, where conserved neural grammars interact with symbolic culture (see 9).

2 Barbieri’s “Code Biology” is a key branch of biosemiotics that studies the rule-based codes underlying biological systems. Just as the genetic code specifies how nucleotide triplets map to amino acids, other biological codes (e.g., splicing codes, signal–response codes) define relationships between cues and functional programs. This framework positions biology as governed not only by chemistry and physics but also by symbolic coding systems that enable meaning and interpretation within living organisms (18–19, 35).

3 The lordosis model is considered canonical because it is one of the most extensively studied examples of a fixed action pattern in mammals: a hormonally primed female reliably displays a stereotyped receptive posture when key stimuli are present. The neural substrates are well characterized—the ventromedial hypothalamus, periaqueductal gray, and medullary reticular formation—making it a benchmark paradigm for linking endocrine drive, conserved circuitry, and contextual gating. If any of these constraints are absent, lordosis fails to occur, abolishing the behavior (20).

4 Freud, Jung, and Lorenz were all physicians (M.D.s). Each brought clinical or biological training to their theories: Freud as a neurologist and psychiatrist, Jung as a psychiatrist, and Lorenz as a physician and ethologist. Their medical grounding shaped their efforts to integrate biology, psychology, and culture—an integrative spirit that we continue to apply in ethology to psychiatry through the ARCH × Φ framework.

5 FAAPs extend Lorenz’s notion of fixed action patterns into the human symbolic domain: they are conserved archetypal scripts that, once activated, unfold in predictable sequences. Unlike animal FAPs, FAAPs are not triggered solely by sensorimotor cues but also by symbolic releasers—such as narratives, images, or ideological frames—which can amplify or distort their expression. This allows ancient neural grammars (e.g., aggression, caregiving, sacrifice) to be rigidly mobilized in cultural and forensic contexts, sometimes producing maladaptive or violent outcomes (see 9).

6 Konrad Lorenz’s classic studies in the 1930s–1950s demonstrated filial imprinting in precocial birds such as geese and ducks. Hatchlings exposed during a critical window shortly after birth would form an attachment to the first moving object they encountered—whether their mother, a human experimenter, or even a toy. Once established, this attachment proved to be long-lasting and difficult to reverse. Imprinting became a foundational model in ethology for experience-dependent, yet evolutionarily conserved, learning, and later informed Bowlby’s attachment theory in psychiatry (37). It is also essential to note that Lorenz was a member of the Nazi Party during World War II; these affiliations have been widely condemned, and our reference to his work is restricted solely to its enduring ethological contributions (42).

7 Entropy in neuroscience often refers to uncertainty—multiple brain states or behavioral scripts competing at once. In hunger, this might mean Navigia (exploring for food), Hedonix (seeking comfort), or even Phobon (defending food resources) are all active in parallel. Crossing threshold (Φ) reduces entropy by collapsing these possibilities into one coherent act—eating—much like finally choosing a TV channel resolves the noise of flipping between many.

8 In computational neuroscience, an *exponential kernel* is a mathematical weighting function that gives greater influence to recent actions, while the impact of earlier actions decays exponentially over time. This provides a compact way to model how Drive (D) integrates prior behavior with a recency bias, consistent with observed learning and adaptation processes in neural systems.

9 In practice, Φ is not directly observed but inferred from behavioral and physiological measures. Candidate indices include: (1) behavioral thresholds such as response latency, frequency, or probability of script activation; (2) physiological correlates such as arousal markers (skin conductance, heart-rate variability), cortisol or neurosteroid levels; and (3) neuroimaging markers such as prefrontal–limbic gating or functional connectivity shifts under symbolic primes. These measures provide testable proxies for when a latent script crosses into overt expression.

10 BADE (*Bias Against Disconfirmatory Evidence*) refers to a cognitive bias in which individuals discount or ignore information that contradicts their existing beliefs. It is commonly studied with experimental tasks presenting ambiguous scenarios that accumulate disconfirmatory cues; high-BADE individuals persist in their initial interpretation despite contradictory evidence. In the ARCH × Φ framework, BADE serves as a proxy for symbolic distortion, where cultural coding rigidly biases script selection against updating.
